# Disproportionately higher cardiovascular disease risk and incidence with high fructose corn syrup sweetened beverage intake among black young adults–the CARDIA study

**DOI:** 10.1186/s12937-024-00978-6

**Published:** 2024-07-29

**Authors:** Luanne Robalo DeChristopher, Katherine L. Tucker

**Affiliations:** 1 Independent Researcher and Guest Researcher, University of Massachusetts Lowell, Lowell, MA USA; 2 Department of Biomedical and Nutritional Sciences and Center for Population Health, University of Massachusetts Lowell, Lowell, MA USA

**Keywords:** African Americans, Heart disease, High fructose corn syrup, Fructose, Fruit drinks, Dysbiosis, Microbiome, Glycation, AGE, FruAGE, Excess-free-fructose, Fructositis, Fructose malabsorption, Race disparity, Hypertension, Unpaired-free-fructose, Apple juice

## Abstract

**Background:**

The black/white heart disease mortality disparity began increasing in the early 1980’s, coincident with the switch from sucrose to high-fructose-corn-syrup/(HFCS) in the US food supply. There has been *more fructose in HFCS than generally-recognized-as-safe/GRAS*, which has contributed to unprecedented excess-free-fructose/(unpaired-fructose) in foods/beverages. Average- per-capita excess-free-fructose, from HFCS, began exceeding dosages/(5-10 g) that trigger fructose-malabsorption in the early 1980’s. Fructose malabsorption contributes to *gut-dysbiosis* and *gut-in-situ-fructosylation* of dietary peptides/incretins/(GLP-1/GIP) which forms atherosclerotic advanced-glycation-end-products. Both dysregulate gut endocrine function and are risk factors for cardiovascular disease/(CVD). Limited research shows that African Americans have higher fructose malabsorption prevalence than others. CVD risk begins early in life.

**Methods:**

Coronary-Artery-Risk-Development-in-Adults/(CARDIA) study data beginning in 1985–86 with 2186 Black and 2277 White participants, aged 18–30 y, were used to test the hypothesis that HFCS sweetened beverage intake *increases CVD risk/incidence*, more among Black than White young adults, and at lower intakes; while orange juice-a low excess-free-fructose juice with comparable total sugars and total fructose, but a 1:1 fructose-to-glucose-ratio, i.e., low excess-free-fructose, does not. Cox proportional hazards models were used to calculate hazard ratios.

**Results:**

HFCS sweetened beverage intake was associated with higher CVD risk (HR = 1.7) than smoking (HR = 1.6). CVD risk was higher at lower HFCS sweetened beverage intake among Black than White participants. Intake, as low as 3 times/wk, was associated with twice the CVD risk vs. less frequent/never, among Black participants only (HR 2.1, 95% CI 1.2–3.7; *P* = 0.013). Probability of an ordered relationship approached significance. Among Black participants, CVD incidence jumped 62% from 59.8/1000, among ≤ 2-times/wk, to 96.9/1000 among 3–6 times/wk consumers. Among White participants, CVD incidence increased from 37.6/1000, among ≤ 1.5-times/wk, to 41.1/1000, among 2 times/wk–once/d – a 9% increase. Hypertension was highest among Black daily HFCS sweetened beverage consumers.

**Conclusion:**

The ubiquitous presence of HFCS over-the-past-40 years, *at higher fructose-to-glucose ratios than generally-recognized-as-safe*, may have contributed to CVD racial disparities, due to higher fructose-malabsorption prevalence among Black individuals, unpaired/excess-free-fructose induced gut dysbiosis and gut fructosylation of dietary peptides/incretins (GLP-1/GIP). These disturbances contribute to atherosclerotic plaque; promote incretin insufficiency/dysregulation/altered satiety/dysglycemia; decrease protective microbiota metabolites; and increase hypertension, CVD morbidity and mortality.

## Introduction

Sugar sweetened beverage (SSB) consumption, i.e., high fructose corn syrup (HFCS) sweetened soda, and fruit drinks, has been associated with cardiovascular disease (CVD) [1–7]. CVD is the leading cause of death in the US [8, 9] and was associated with higher COVID-19 death rates [10]. Proposed mechanisms thought to link SSB with heart disease include effects of unregulated, unchecked fructose metabolism by the liver, which increases triglycerides (TG), cholesterol, uric acid, and visceral and ectopic fat. Onset of three of five risk factors (hyperglycemia, hypertension, hypertriglyceridemia, dyslipidemia, and central adiposity), increases type 2 diabetes (T2D) and CVD risk/incidence [11]. However, these pathways do not fully explain the SSB/heart disease association in Black individuals, who are 30% more likely to die from heart disease than non-Hispanic White individuals – a disparity which began inexplicably increasing in the US in the early 1980s [12, 13]. Serum TG, which should rise due to unregulated, unchecked fructose metabolism, are often *normal* in the presence of T2D and CVD in Black individuals [14, 15], and they are more likely to be insulin *insufficient* than insensitive [15]. Therefore, other risk factors underlie the Black/White CVD mortality disparity.

The early 1980’s [16–19] coincides with the shift from sucrose to HFCS in US soft drinks (~ 1980–1984) and its proliferation throughout the US food supply [18, 19]. In the early 1980’s, average per capita excess-free-fructose/unpaired fructose intake from HFCS *began exceeding dosages (5–10 g) that trigger fructose malabsorption* [20]–an overlooked CVD risk factor in the emerging fructose/gut/heart axis [21–45]. HFCS became the cheaper alternative due to high import tariffs on sucrose [46, 47], and because it contains more fructose than sucrose. Higher fructose translates to lower costs/higher profits, as fructose is ~ twice as sweet as glucose. Less is needed to achieve targeted sweetness [48]. Fructose malabsorption [49–59] occurs after intake of sugars with high fructose-to-glucose ratios, i.e. unpaired fructose/excess-free-fructose, as in HFCS [60, 61], apple juice/powder [62], agave syrup (70%-90% fructose) [63], and crystalline fructose, but *not* sucrose or paired fructose/glucose which occurs naturally in orange juice [62].

Limited research shows that Black individuals have *higher* fructose malabsorption prevalence at lower unpaired fructose intake than other groups [64]. Fructose is more readily absorbed when paired with glucose [55]. Unpaired fructose that is not absorbed *is not* metabolized by the liver. It *will not* contribute to TG production, hypertriglyceridemia, or metabolic hyperuricemia. On the other hand, unabsorbed unpaired fructose, promotes *gut* in situ chemical modification (fructosylation) of partially digested dietary proteins and gut hormones (GLP1/GIP), an overlooked source of atherogenic advanced glycation end-products (AGE), referred to as FruAGE [65–70], and gut hormone dysregulation [68, 70]. High FruAGE burden contributes to *disproportionately higher serum AGE* to soluble AGE receptors (sRAGE), as observed in Black adults [69]. AGE that outnumber sRAGE are proinflammatory and atherogenic, because sRAGE are “ligand decoys” that neutralize atherosclerotic AGE [69]. Unabsorbed unpaired fructose is also associated with gut dysbiosis [21, 25, 49, 50, 71]–a condition characterized by lower gut microbiome diversity, altered bacterial composition, and an altered metabolome. Research shows that the gut microbiome plays an important role in the development/pathogenesis of chronic diseases [21–45] including hypertension [27–30], dyslipidemia [31, 32], hyperuricemia [33–35], kidney disease [36–38], respiratory disease [20], hyperinsulinemia [45], systemic inflammation, and CVD [21–45]. Fructose in the gut elevates lipopolysaccharides (LPS) [71]. FruAGE and LPS activate RAGE signaling which is associated with increased CVD mortality [69–73]. Gut dysbiosis also impedes the normal functioning of GIP and GLP-1 [45]. Their disruption promotes weight gain, insulin *insufficiency* and hyperglycemia–CVD risk factors [45]. Outward symptoms (gas, bloating, and abdominal pain) are often lacking in fructose malabsorption [74]. Importantly, independent labs measured the fructose content in the HFCS in popular soft drinks and found that it contains *higher* fructose-to-glucose ratios (1.9:1 [60] and 1.5:1 [61]) than generally-recognized-as-safe (GRAS) (1.2:1) [75] which poses significantly greater risks to fructose malabsorbers [49–59]. Epidemiological studies of HFCS sweetened beverage intake and heart disease among Black adults are lacking.

### Study objectives

CVD risk begins early in life [76]. Therefore, we aimed to test the hypothesis that Black young adults who regularly consume HFCS-sweetened beverages, *during young adulthood,* have increased (fatal/non-fatal) CVD risk/incidence, independent of potential confounders at enrollment, including hypertriglyceridemia, hyperuricemia, hypertension, dyslipidemia, hyperglycemia, overweight, smoking, physical activity, and dietary factors, including fast food intake frequency, and that *CVD risk/incidence increases with increasing intake and* is *higher at lower intakes among Black individuals*.

We hypothesized that regular intake of orange juice may be protective against CVD. Orange juice has comparable total sugars, total fructose, and a similar glycemic load, as non-diet cola and apple juice, but unlike them, OJ contains a ~ 1:1 fructose-to-glucose ratio [20, 62, 77], i.e., nominal excess-free-fructose, and *is not* associated with fructose malabsorption.

## Methods

### Study design and potential confounders

Survival analysis was conducted with data from the Coronary Artery Risk Development in Young Adults (CARDIA) Study [78], a longitudinal study aimed at investigating the development and determinants of clinical and subclinical CVD and their risk factors. CARDIA participants were selected to have approximately the same numbers in subgroups of race (Black and White), sex, education and age (18–30 y) in each of 4 centers: Birmingham, AL; Chicago, IL; Minneapolis, MN; and Oakland, CA (*n* = 5115). To test our hypothesis, we conducted survival analysis with prospective data from Black and White participants (4500), with non-missing demographic, dietary, and lifestyle data, from enrollment (1985–86) through approximately 35 years of follow-up. Survival analysis was conducted by race with data collected at enrollment.

The CARDIA Study was well suited to test our hypothesis, as peak adult soft drink/fruit drink consumption occurs between the ages of 18 – 39 y [79], i.e., coincident with the average age of participants at enrollment (24.5 y), and enrollment occurred in 1985–86, after the switch from sucrose to HFCS (1980–1984) in US soft drinks/fruit drinks [16–19, 46, 47]. Survival analysis was conducted using Cox regression models (1–3). No participant at enrollment had a history of one or more clinical CVD events, as described elsewhere [80].

Non-missing medical laboratory data collected at enrollment were also included as potential confounders. Of the Black / White CARDIA participants who enrolled in the study, there were the following exclusions: 363 / 131 due to implausible energy intake, defined as mean total daily energy intake ≤ 600 or ≥ 5000 kcal, 88 / 66 due to missing variables in Cox regression Models 1, and 1 / 4 exclusions, respectively, due to missing variables in Cox regression Models 2 and 3. After exclusions, there were 2186 (Model 1), and 2185 (Models 2 and 3) Black participants, and 2277 (Model 1), and 2273 (Models 2 and 3) White participants in the analysis. Lifestyle (smoking status/history, physical activity), dietary, and physiological CVD risk factors were also analyzed by beverage intake frequency.

We also conducted the Chi-square Test for Homogeneity of participants lost to follow-up by HFCS sweetened beverage intake and race, as we were interested in assessing potential differences, given that contributions to time-on-study by individuals lost-to-follow-up can introduce a margin of error into analysis results, particularly when loss-to-follow-up occurs disproportionately by exposure (HFCS sweetened beverage intake frequency) and group (race).

The CARDIA Study is supported and funded by the National Heart, Lung, and Blood Institute (NHLBI) of the National Institutes of Health (NIH) [78]. This analysis was approved/received exempt status by the Institutional Review Board of the University of Massachusetts Lowell.

### Beverage intake

We analyzed intake frequency of HFCS sweetened beverages (non-diet soda and fruit drinks), which have been shown to contain high excess-free-fructose (EFF)/unpaired fructose concentrations, higher than generally-recognized-as-safe [60, 61]. Coca Cola®^©^ has a glycemic load (GL) of 16 / 250 ml [77]. Cola contains 26 g total sugars, 16 – 17 g of total fructose [62], and 5—9 g of EFF / 250 ml, [20] i.e., as based upon fructose-to-glucose ratios measured by independent labs (1.5:1 and 1.9:1) [60, 61]. We also analyzed intake frequency of 100% orange juice, a juice with a similar glycemic load (15 / 250 ml) [77], total sugars (21 g / 250 ml), and total fructose (11 g / 250 ml) as cola and apple juice, but *unlike* them, OJ has a ~ 1:1 fructose-to-glucose ratio, i.e., nominal excess-free-fructose (0.4 g EFF / 250 ml) [62]. Apple juice is a primary juice in HFCS sweetened fruit drinks. Its glycemic load (12 / 250 ml), total sugars (24 g / 250 ml), and total fructose (16 g / 250 ml) are comparable to cola and 100% orange juice, but *like* HFCS, it contains high excess-free-fructose (8 g EFF / 250 ml) [62, 77]. Lastly, we *did not* analyze “100% non-citrus juices” as intake of high EFF apple juice, was not distinguished from other non-citrus juices, with low/nominal excess-free-fructose (grape (1.4 g EFF), pineapple (2.1 g EFF / 250 ml) and others) [62]. Results would be difficult to interpret.

Beverage intake data were obtained via a diet history questionnaire administered at enrollment (exam 1). CARDIA participants were asked, “Do you usually drink any fruit or vegetable juices? Do you usually drink Coke, soda, or pop? How much do you usually have? How often?” Responses were distinguished by sweetener type (diet vs. non-diet). Volume was provided by participants as cups or ounces, and intake frequency as daily, monthly, or weekly [78]. The data*, as provided* by CARDIA, were normalized to cups/d. Intake of any combination of HFCS sweetened beverages and 100% citrus juice was divided into ordered quintiles (whole cohort) and ordered quartiles (analysis by race) for Cox regression analysis. Nutrient analyses from the CARDIA Study showed that the dietary history provided estimates that agreed reasonably well with expected energy intake for body mass index (BMI), according to the age/sex-specific Recommended Dietary Allowances [78]. This is consistent with research which found good correlation between frequency of food and food group consumption and probability of consumption on 24-h dietary recalls [81].

### Ascertainment of endpoints

Incident CVD was defined as the first event of definite or probable (fatal/non-fatal) coronary heart disease (CHD); including myocardial infarction (MI), angina pectoris, and death due to CHD, stroke, transient ischemic attack, heart failure, or peripheral artery disease through approximately 35 follow up years. CVD events were adjudicated by a team of experts who used published guidelines, as described in detail elsewhere [78].

### Risk assessments and potential confounders

Three Cox proportional hazards models, with time in the study as the time scale, were used for analysis. Proportional hazards assumptions were assessed using Schoenfeld and scaled Schoenfeld residuals for the models (*P* ≥ 0.05), and via Kaplan Meier survival curve plots for each predictor. We examined incident CVD over approximately 35 y of follow-up using multivariable adjusted Cox proportional hazards models to estimate hazard ratios (HR). Person-time was calculated from enrollment (1985–86) through follow-up (~ 35 y), loss to follow-up, death from causes other than CVD, incident fatal/non-fatal CVD, whichever came first. R and Rstudio version 2022.07.2 were used and a two-tailed *P* value ≤ 0.05 with 95% confidence intervals (CI) that did not include 1, were considered statistically significant.

Potential confounders were selected based on existing research [1–5]. Three models were used to analyze variables that affected the association between beverage intake and CVD. Cox regression Models 1—3 include potential confounders collected at exam 1 / enrollment. Model 1 includes the following potential confounders: sex; total energy intake (kcal); education in years (continuous); age (18–25 or 25–30 y); body mass index (BMI); hypertension status obtained from the average of 2 cuff readings (Normal—SBP < 120 and DBP < 80 mmHg; Stage 1 hypertension (SBP ≥ 120 -139 or DBP ≥ 80—89 mmHg); or Stage 2 hypertension (SBP ≥ 140 or DBP ≥ 90 mmHg)); self-reported hypertension or use of hypertension medicine (Y/N). Models 1–3 also included the following potential confounders collected at enrollment: serum concentrations of LDL-C, triglycerides, uric acid, apoB lipoproteins, insulin, and plasma glucose.

Model 2 was further adjusted for smoking status (never, or past/current smoker) and when missing, responses were updated with answers provided during exam 2; physical activity history score [82]; combined daily fruit and vegetable intake, normalized to servings/d; fast food visit frequency, normalized to frequency of visits/wk, asked as “How often do you eat breakfast, lunch or dinner out in a place such as McDonalds, Burger King, Wendys, Arbys, Pizza Hut, or Kentucky Fried Chicken”; and alcohol intake (servings/d in quartiles). Model 3 was further adjusted for intake of other beverages (continuous variables). For example, orange juice analysis included HFCS sweetened beverage and 100% non-citrus juice intake.

**Fig. 1:**
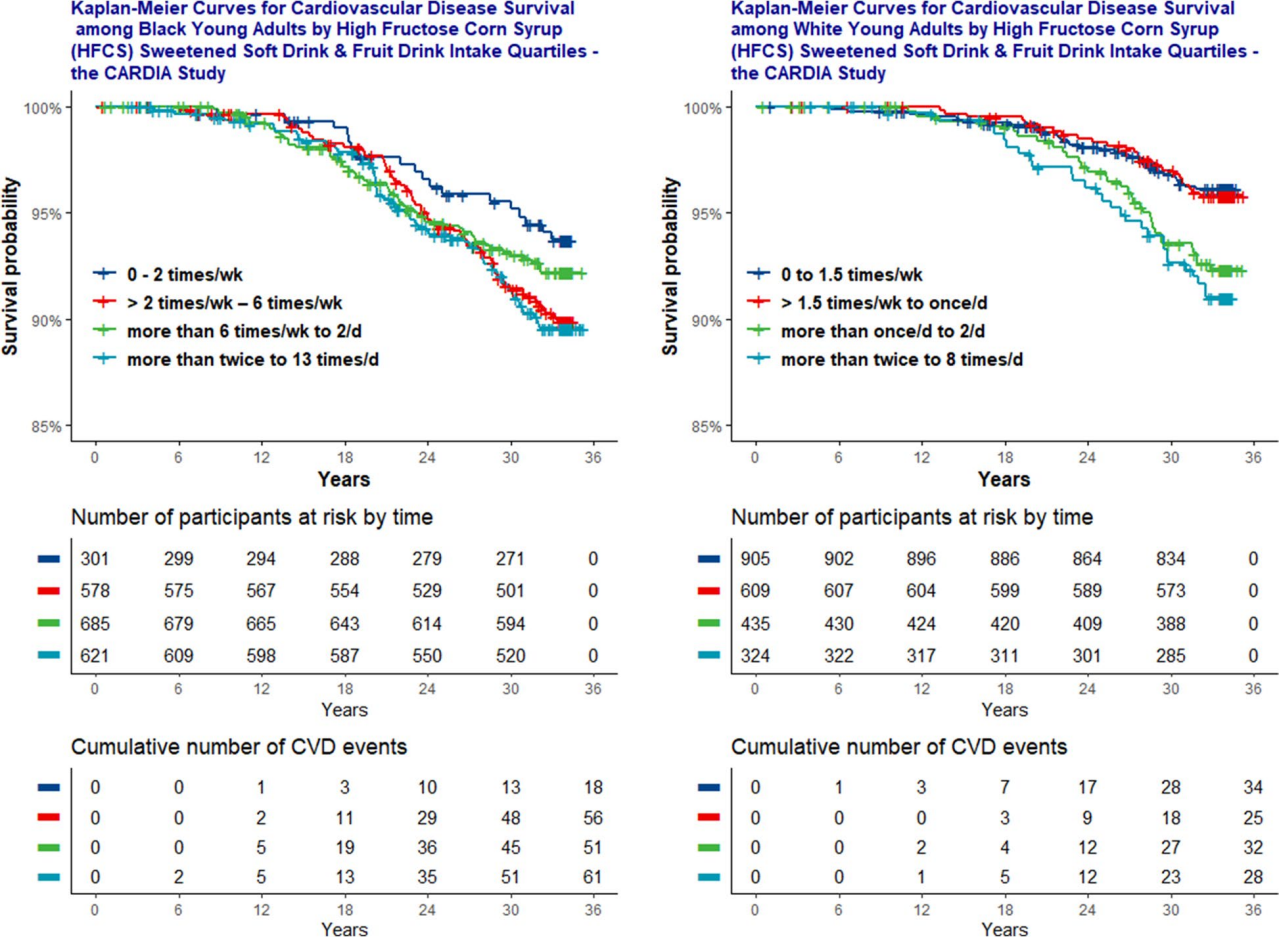
Kaplan-Meier curves for cardiovascular disease survival among young adults by high fructose corn syrup (HFCS) sweetened beverage intake and race

Kaplan Meier Curves are included which depict CVD Survival Probability over time (35 y), by HFCS sweetened beverage intake frequency (quartiles), and race. Plots include “Number of Participants at Risk by Time” and “Cumulative Number of CVD Events by Time”, Fig. 1.

## Results

### Baseline characteristics of participants

The mean age of participants was 24.5 y; 60% and 55% of Black and White participants were female; 56% and 78% were at recommended weight; 32% and 26% were current smokers; and 52% and 75% attended / completed post-secondary education, respectively. Black participants consumed HFCS sweetened beverages (56.2% ≥ once/d) and 100% orange juice (40.1% ≥ once/d) more frequently than White participants (31.1% and 33.2% ≥ once/d). Rates of pre-diabetes, T2D, dyslipidemia, hyperuricemia, and high-risk fasting serum Apolipoprotein B to Apolipoprotein A1 Ratios were comparable across participants, but not hypertriglyceridemia and hypertension. Hypertriglyceridemia was higher among White (7.2%) than Black participants (3.1%), even though fewer (30.7%) consumed HFCS sweetened beverages multiple times/d than Black participants (56.2%). Stage 1 and stage 2 hypertension and hyperinsulinemia were higher among Black participants (24.5% / 8.2%) than White participants (20.7% / 2.5%). Data not shown.

**Fig. 2:**
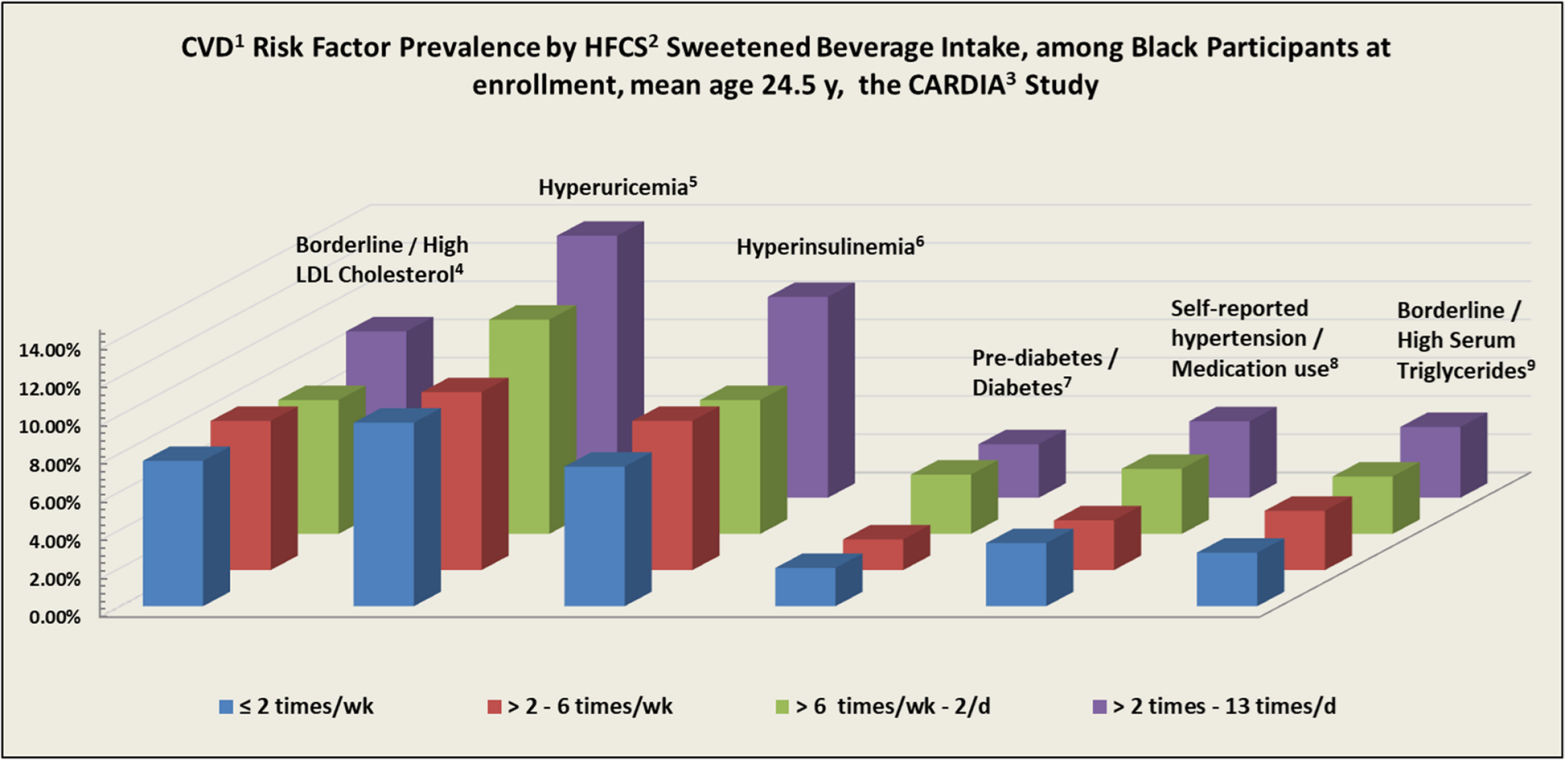
^1^CVD—cardiovascular disease. ^2^High fructose corn syrup. ^3^Coronary Artery Risk Development in Young Adults. ^4^Borderline / High LDL – Cholesterol (≥ 190 mg/dL) at enrollment. ^5^Hyperuricemia (> 7.0 mg/dL men, > 6.0 mg/dL women) at enrollment. ^6^Hyperinsulinemia (≥ 25 mcU/ML) at enrollment. ^7^Hyperglycemia—Prediabetes / Diabetes (≥ 100–125 mg/dL) at enrollment. ^8^Self-reported Hypertension / Hypertension medication-use at enrollment. ^9^Borderline / High Serum Triglycerides (Borderline High (150–199 mg/dL / High (≥ 200 mg/dL))

Daily total energy intake and physical activity *increased* with *increasing* consumption across beverages. Smoking prevalence was highest among multiple times/d HFCS sweetened beverage consumers, (45.7% (Black) and 54.9% (White)). Among Black participants, hypertriglyceridemia (0.3 – 1.4%), hyperuricemia (9.6 – 13.7%), hyperglycemia (1.3 – 2.3%), hyperinsulinemia (7.3 – 10.5%), hypercholesterolemia (7.6 – 8.7%), and hypertension (2.6 – 4%) *increased* with *increasing* consumption of HFCS sweetened beverages, Table 1 and Fig. 2. Increasing patterns were also seen among White participants (2.0 – 4.6%) / (9.5 – 20.4%) / (1.7 – 4%) / (1.4 – 4.0%) / (5.1 – 7.1%), but not hypertension which was *lowest* among White *daily* HFCS consumers (1.8 – 0.6%). Notably, elevated serum TG and uric acid were *highest* among White participants even though they consumed HFCS sweetened beverages *less often* than Black participants Table 2. Also notable is that Black ≥ 2 times/wk HFCS sweetened beverage consumers, i.e., moderate consumers, had *higher* hyperinsulinemia (7.3%) than White *multiple times/d* consumers (4.0%).

**Table 1:** Characteristics of black participants by beverage intake frequency at enrollment, the CARDIA^a^ study

		Any Combo of HFCS^b^ sweetened Soft Drinks and Fruit Drinks				100% Citrus / Orange Juice^c^			
Overall No. = 2185	No.	301	578	685	621	545	544	541	555
**Intake Frequencies**		** ≤ 2 times/wk**	** > 2/wk -6/wk**	** > 6/wk—twice/d**	** > twice/d -13/d**	** ≤ twice/wk**	**3/wk** **5/wk**	** > 5/wk -1.5/d**	** > 1.5/d** **6/d**
Normalized to 1/cup									
**Education Years** (Mean) (SD)		13.5 ± 1.8	13.3 ± 1.8	13.1 ± 1.7	12.8 ± 1.6	12.9 ± 1.7	13.1 ± 1.7	13.3 ± 1.8	13.2 ± 1.7
**Active / former smoker (%)**		39.7	36.7	41.0	45.7	45.0	42.1	39.9	37.3
**BMI (**kg/m**) (**Mean**)** (SD)		26.0 ± 5.6	25.1 ± 5.1	25.3 ± 5.7	25.6 ± 5.7	25.4 ± 5.6	25.5 ± 6.0	25.0 ± 5.0	25.7 ± 5.5
**Energy (kcal) (**Mean**)** (SD)		2150 ± 983	2360 ± 988	2660 ± 999	3100 ± 978	2300 ± 1010	2510 ± 995	2730 ± 1010	2990 ± 1030
**Fruit & vegetable mean serv/d** (SD)		5.3 ± 3.8	5.2 ± 3.8	5.2 ± 3.6	5.5 ± 4.1	3.3 ± 2.9	4.1 ± 2.7	5.6 ± 3.2	8.2 ± 4.3
**Alcohol servings/d** (Mean) (SD)		0.4 ± 0.9	0.5 ± 1.0	0.6 ± 1.1	0.7 ± 1.2	0.5 ± 1.2	0.6 ± 1.1	0.6 ± 1.0	0.6 ± 1.1
**Frequency/wk of fast-food visits** (SD)		1.6 ± 1.9	1.6 ± 1.6	2.1 ± 1.1	2.2 ± 1.1	1.8 ± 2.0	2.0 ± 2.0	2.0 ± 2.1	2.0 ± 2.0
**Physical Activity Score** (SD)		355 ± 272	351 ± 289	366 ± 299	364 ± 289	295 ± 253	332 ± 272	400 ± 309	411 ± 306
**Self-reported**									
**Hypertension/Med Use (%)**		3.3	2.6	3.4	4.0	2.8	4.0	3.1	3.4
**Serum Triglycerides (%)**									
Borderline High (150–199 mg/dL)		2.0	1.9	2.0	2.3	2.2	1.8	2.2	2.0
High (≥ 200 mg/dL)		0.3	1.2	1.0	1.4	1.1	1.1	0.9	1.3
**Hyperuricemia (%)**									
(> 7.0 mg/dL men, > 6.0 mg/dL women)		9.6	9.3	11.2	13.7	9.0	13.1	12.9	9.9
**Hypercholesterolemia (%)**									
≥ 190 mg/dL		7.6	7.8	7.0	8.7	6.8	7.9	8.1	8.3
**Hyperglycemia (%)**									
Pre-diabetes 100–125 mg/dL		1.3	1.4	1.9	2.3	1.7	1.5	1.8	2.2
Diabetes (≥ 126 mg/dL)		0.7	0.2	1.2	0.5	0.4	0.7	0.9	0.5
**Hyperinsulinemia** (≥ 25 mcU/ML)		7.3	7.8	7.0	10.5	8.6	9.6	6.7	8.1
**Apolipoprotein B to A1 Ratio (%)**									
Higher CVD risk (> 0.9 /men, > 0.8 mg/dL / women)		14.6	15.7	16.5	16.6	14.7	16.7	15.2	17.7

^a^CARDIA Study-Coronary Artery Risk Development in Young Adults Study

^b^HFCS – high fructose corn syrup which contains more excess-free-fructose than safe, i.e., 5 – 9 g /250 ml, as measured by independent labs [20, 60, 61]

^c^Orange juice—a low excess-free-fructose juice (0.4 g / 250 ml)

**Table 2:** Characteristics of white participants by beverage intake frequency at enrollment, the CARDIA^a^ study

		**Any Combo of HFCS**^b^ **sweetened** **Soft Drinks, and Fruit Drinks**				**100% Citrus / Orange Juice**^**c**^			
Overall No. = 2273	No.	905	609	435	324	621	615	573	464
**Intake Frequencies**		** ≤ 1.5/wk**	** > 1.5/wk—once/d**	** > once/d** **2/d**	** > 2/d -8 times/d**	** ≤ 2 times/wk**	** > 2/wk-5 times/wk**	** > 5/wk -1.5/d**	** > 1.5/d-6/d**
**Education Years** (Mean) (SD)		15.0 ± 1.8	14.6 ± 1.9	14.2 ± 2.0	13.3 ± 1.9	14.2 ± 2.1	14.6 ± 2.0	14.6 ± 2.0	14.5 ± 2.0
**Active / former smoker (%)**		40.9	40.4	47.1	54.9	47.5	42.1	43.3	42.5
**BMI (**kg/m**) (**Mean**)** (SD)		23.2 ± 3.6	23.5 ± 3.7	23.8 ± 3.9	24.3 ± 4.3	23.7 ± 4.1	23.6 ± 3.8	23.6 ± 3.7	23.5 ± 3.6
**Energy (kcal) (**Mean**)** (SD)		2140 ± 838	2470 ± 847	2740 ± 955	3150 ± 905	2220 ± 901	2350 ± 864	2630 ± 953	2850 ± 926
**Fruit & vegetable mean serv/d** (SD)		6.3 ± 3.9	6.0 ± 3.5	5.6 ± 3.7	4.9 ± 3.8	4.2 ± 3.1	4.9 ± 3.0	6.4 ± 3.3	8.8 ± 4.1
**Alcohol servings/d** (Mean) (SD)		0.7 ± 0.8	0.8 ± 1.0	1.0 ± 1.3	0.9 ± 1.2	0.7 ± 1.0	0.8 ± 1.0	0.9 ± 1.0	0.9 ± 1.1
**Frequency/wk of fast-food visits** (SD)		1.3 ± 2.0	1.6 ± 1.9	2.1 ± 2.1	3.2 ± 2.0	1.8 ± 2.4	1.8 ± 2.1	1.8 ± 2.3	1.8 ± 2.3
**Physical Activity Score** (SD)		460 ± 273	441 ± 293	439 ± 271	408 ± 275	388 ± 263	434 ± 251	493 ± 312	468 ± 276
**Self-reported**									
**Hypertension/Med Use (%)**		1.3	1.8	1.4	0.6	1.9	1.5	0.9	1.1
**Serum Triglycerides (%)**									
Borderline High (150–199 mg/dL)		2.7	5.3	5.1	6.5	4.7	4.1	3.7	5.2
High (≥ 200 mg/dL)		2.0	2.3	3.9	4.6	3.2	2.3	2.6	3.2
**Hyperuricemia (%)**									
(> 7.0 mg/dL men, > 6.0 mg/dL women)		9.5	11.5	17.0	20.4	12.9	13.0	13.1	13.1
**Hypercholesterolemia (%)**									
≥ 190 mg/dL		5.1	5.6	6.2	7.1	6.4	6.8	4.7	4.5
**Hyperglycemia (%)**									
Pre-diabetes 100–125 mg/dL		1.7	2.6	2.3	4.0	2.4	2.4	2.1	2.6
Diabetes (≥ 126 mg/dL)		0.4	0.2	0.5	0.0	0.5	0.2	0.2	0.4
**Hyperinsulinemia** (≥ 25 mcU/ML)		1.4	2.8	3.0	4.0	4.2	1.3	1.4	3.0
**Apolipoprotein B to A1 Ratio (%)**									
Higher CVD risk (> 0.9 /men, > 0.8 mg/dL / women)		14.5	18.2	20.5	28.7	23.7	18.5	16.1	15.3

^a^CARDIA Study-Coronary Artery Risk Development in Young Adults Study

^b^HFCS – high fructose corn syrup which contains more excess-free-fructose than safe, i.e., 5 – 9 g /250 ml, as measured by independent labs [20, 60, 61]

^c^Orange juice—a low excess-free-fructose juice (0.4 g / 250 ml)

Self-reported hypertension/medicine use *decreased* with *increasing* consumption of 100% orange juice, across participants**.** Hypertriglyceridemia, and hyperuricemia either remained even or *decreased* with *increasing* consumption of 100% orange juice, irrespective of race. Hypercholesterolemia *decreased* with *increasing* consumption of 100% orange juice among White participants only, Tables 1 and 2.

### Loss-to-follow-up

Loss-to-follow-up, herein, defined as participants who did not attend the last exam, was significantly higher among Black (668) than White (479) participants. Of the 668 Black participants lost-to-follow-up, more than half (59%) were daily consumers of HFCS sweetened beverages. Disproportionate loss-to-follow-up, by race and beverage intake frequency, may have contributed to CVD risk and incidence *underestimation* among Black daily consumers of HFCS sweetened beverages (Table 3).

**Table 3:** Loss to follow-up by high fructose corn syrup sweetened beverage intake quartile and race

HFCS sweetened beverage intake quartile	Race	Loss-to-follow-up (Y) # of Participants	Loss-to-follow-up (N) # of Participants	Total	Chi Square Test for Homogeneity *P*-value
**≤ 2 times/wk**	Black	89 (29.5%)	213	302	
**≤ 1.5 times/wk**	White	174 (19.1%)	737	911	0.0002^***^
**> twice/wk – 6 times/wk**	Black	184 (31.7%)	396	580	
**> 1.5 times/wk – once/d**	White	120 (19.5%)	495	615	1.770459e-06^***^
**> 6 times/wk – twice/d**	Black	191 (27.6%)	501	692	
**> once/d – 2 times/d**	White	102 (23.3%)	336	438	0.123
**> times/d – 13 times/d**	Black	204 (32.5%)	424	628	
**> 2 times/d – 8 times/d**	White	83 (25.2%)	247	330	0.0226^*^

^*^Asterisk indicates statistical significance

^***^Multiple asterisks indicate high statistical significance

### CVD incidence

There were 186 new CVD cases among Black (*n* = 2186) and 119 new CVD cases among White (*n* = 2277) CARDIA participants, over ~ 35 follow-up years.

### Relationship with CVD

HFCS associations with CVD were significant among Black participants only. Black participants who consumed HFCS sweetened beverages 3 – 6 times/wk and multiple times/d, had 2 times higher CVD risk, relative to ≤ 2 times/wk consumers, independent of confounders (HR = 2.1, 95% CI 1.2–3.7, *P* = 0.01) / (HR = 2.0, 95% CI 1.1–3.6, *P* = 0.03). *P* for trend approached significance (*P* = 0.08). Loss-to-follow-up bias plausibly explains the comparable two-fold higher CVD risk among 3 – 6 times/wk consumers, and multiple times/d consumers. Lack of a stepwise increase in CVD risk (hazard ratios) with increasing intake, among Black participants, may be attributable to loss-to-follow-up bias. Nonetheless, CVD incidence increased from 59.8/1000, among ≤ 2 times/wk HFCS sweetened beverage consumers, to 98.2/1000 among multiple times/d – a 64% increase*.* Incident cases/1,000/y increased with increasing intake, from 1.9/1000/y among less frequent/never (≤ 2 times/wk) to 3.1/1000/y among multiple times/d consumers – a 63% increase, Table 4. There was *an opposite trend* with orange juice intake. Among Black participants, CVD incidence per 1000 and number of cases per 1000/y *decreased with increasing* orange juice intake, from 99.3/1000 among > 2—5 times/wk to 79.3/1000 among *daily* orange juice consumers – a 25% *decrease*, and from 3.1/1000/y to 2.5/1000/y – a 19% decrease, Table 4.

**Table 4:** Cardiovascular disease^b^ risk according to beverage consumption among black participants, the CARDIA study^c^

Cox Proportional Hazards Hazard Ratios (HR)	No.	HR	Model 1 95% CI	*P*-value	*P* for Trend	HR	Model 2 95% CI	*P*-value	*P* for Trend	HR	Model 3 95% CI	*P*-value	*P* for Trend	No. Cases	IR per (1000)	Person Time Years	Cases P/1000 P/y
**any combination of HFCS**^**d**^** sweetened non-diet soda and fruit drinks**		**HR**– **adjusted for age**^**e**^**, sex, total energy intake, education, BMI, hypertension, LDL-C,**^**f**^** hypertension medicine use, TG,**^**g**^** uric acid, apoB,**^**h**^** insulin, and plasma glucose concentration**				**HR– further adjusted for physical activity, alcohol, fruit, vegetable intake frequency, frequency/wk of fast-food visits, smoking status (active/former vs. never)**				**HR-further adjusted for intake of other beverages**^**a**^							
		*n* = 2186				*n* = 2185				^**a**^**adjusted for 100% citrus and non-citrus juice intake** *n* = 2185							
** ≤ 2 times/wk**	301	Reference -------				Reference -------				Reference -------				18	59.8	9714	1.9
** > 2 – 6 times/wk**	578	1.98	1.12 – 3.51	0.019^*^		2.05	1.16 – 3.65	0.014^*^		2.07	1.16 – 3.67	0.013^*^		56	96.9	18563	3.0
** > 6/wk – 2/d**	685	1.38	0.77 – 2.48	0.279		1.44	0.80 – 2.60	0.219		1.45	0.81 – 2.61	0.210		51	74.5	21780	2.3
** > 2 – 13 times/d**	**621**	**1.84**	**1.01 – 3.32**	0.045^*^	**0.12**	**1.92**	**1.05 – 3.50**	0.033^*^	**0.09 ~ **	**1.96**	**1.08 – 3.57**	0.028^*^	**0.08 ~ **	**61**	**98.2**	**19531**	**3.1**
**100% Citrus Juice (Orange Juice)**^**i**^										^**a**^**adjusted for intake of any combination of HFCS**^**d**^** sweetened beverages and 100% non-citrus juices**							
** ≤ 2 – times/wk**	545	Reference -------				Reference -------				Reference -------				46	84.4	17435	2.6
** > 2 – 5 times/wk**	544	1.10	0.73 – 1.66	0.641		1.17	0.77 – 1.77	0.459		1.16	0.77 – 1.77	0.478		54	99.3	17318	3.1
** > 5/wk – 1.5/d**	541	0.86	0.55 – 1.35	0.508		0.91	0.57 – 1.43	0.676		0.94	0.60 – 1.47	0.780		42	77.6	17151	2.4
** > 1.5—8/d**	555	0.87	0.57 – 1.33	0.521	0.33	0.95	0.60 – 1.49	0.815	0.57	0.93	0.58 – 1.48	0.752	0.56	44	79.3	17683	2.5

^*^Asterisks indicate statistical significance

~ This symbol indicates results approached statistical significance

^a^beverages that were included in model 3 analyses

^b^CVD—was defined as the first event of definite or probable (fatal/non-fatal) coronary heart disease (CHD); including myocardial infarction (MI), angina pectoris, and death due to coronary heart disease (CHD), stroke, transient ischemic attack, heart failure, or peripheral artery disease from 1985 (mean age 24.5 y) through approximately 35 years of follow-up

^c^CARDIA Study—Coronary Artery Risk Development in Young Adults Study

^d^HFCS – high fructose corn syrup which contains more excess-free-fructose and higher fructose-to-glucose ratios than safe, as measured by independent labs, i.e., 5 – 9 of unpaired / excess-free-fructose g / 250 ml [20, 60, 61]

^e^Age – a 2-level variable defined as 18–24 and 25–30 y

^f^Low density lipoprotein cholesterol (LDL-C) serum concentration – a continuous variable

^g^Serum triglyceride concentration (a continuous variable)

^h^Serum apolipoprotein B concentration (a continuous variable)

^i^Orange juice—a low excess-free-fructose juice (0.4 g / 250 ml)

Among Black participants, obesity, current/former smoker, hypertriglyceridemia, hyperglycemia, hyperuricemia, and hypertension were significantly associated with increased CVD risk, independent of confounders, and higher education appeared protective. The highest CVD risks were with self-reported hypertension / med use (HR 2.3, 95% CI 1.3 – 4.0, *P* = 0.002). In a fully adjusted model, CVD risk rose 12% per unit increase in HFCS sweetened beverage consumption (HR 1.12, 95% CI 1.03–1.2, *P* = 0.009), (data not shown).

Among White participants, none of the beverages, when analyzed by intake quartile, were associated with increased CVD risk. However, CVD incidence (37.6/1000 – 86.4/1000) and cases per 1000/y (1.1/1000/y – 2.7/1000/y) increased 130% and 145% respectively, with increasing HFCS sweetened beverage consumption and *decreased with increasing* consumption of orange juice (66/1000 – 51.7/1000), Table 5. Among White participants, hypertension, hypercholesterolemia, hyperglycemia and age at enrollment were associated with increased CVD risk, independent of confounders. The highest CVD risks were with BP cuff readings consistent with stage 2 hypertension (HR 2.6, 95% CI 1.1 – 6.2, *P* = 0.03). In a fully adjusted model, CVD risk rose 14% per unit increase in HFCS sweetened beverage consumption (HR 1.14, 95% CI 1.01 – 1.3, *P* = 0.034) (data not shown).

**Table 5:** Cardiovascular disease^b^ risk according to beverage consumption among white participants, the CARDIA study^c^

Cox Proportional Hazards Hazard Ratios (HR)	No.	HR	Model 1 95% CI	*P*-value	*P* for Trend	HR	Model 2 95% CI	*P*-value	*P* for Trend	HR	Model 3 95% CI	*P*-value	*P* for Trend	No. Cases	IR per (1000)	Person Time Years	Cases P/1000 P/y
**any combination of HFCS**^**d**^** sweetened non-diet soda and fruit drinks**		**HR**– **adjusted for age**^**e**^**, sex, total energy intake, education, BMI, hypertension, LDL-C,**^**f**^** hypertension medicine use, TG,**^**g**^** uric acid, apoB,**^**h**^** insulin, and plasma glucose concentration**				**HR– further adjusted for physical activity, alcohol, fruit, vegetable intake frequency, frequency/wk of fast-food visits, smoking status (active/former, vs. never)**				**HR-further adjusted for intake of other beverages**^**a**^							
		*n* = 2277				*n* = 2273				^**a**^**adjusted for 100% citrus and non-citrus juices** *n* = 2273							
** ≤ 1.5 times/wk**	905	Reference -------				Reference -------				Reference -------				34	37.6	29743	1.1
** > 1.5 /wk – once/d**	609	0.91	0.53 – 1.55	0.724		0.88	0.51 – 1.50	0.634		0.88	0.52 – 1.50	0.646		25	41.1	20151	1.2
** > 1/d – twice/d**	435	1.45	0.86 – 2.43	0.165		1.32	0.78 – 2.21	0.301		1.31	0.78 – 2.20	0.310		32	73.6	14079	2.3
** > twice/d—8/d**	324	1.58	0.90 – 2.77	0.113	0.04	1.28	0.72 – 2.27	0.401	0.21	1.28	0.73 – 2.26	0.394	0.22	28	86.4	10438	2.7
**100% Citrus Juice (Orange Juice)**^**i**^										^**a**^**adjusted for intake of any combination of HFCS**^**d**^** sweetened Beverages and 100% non-citrus juices**							
** ≤ 2 – times/wk**	621	Reference -------				Reference -------				Reference -------				41	66.0	20193	2.0
** > 2 – 5 times/wk**	615	0.60	0.36 – 0.99	0.048^*^		0.66	0.40 – 1.11	0.120		0.69	0.41 – 1.15	0.157		25	40.7	20164	1.2
** > 5/wk – 1.5/d**	573	0.80	0.48 – 1.34	0.400		1.03	0.61 – 1.74	0.919		1.04	0.61 – 1.77	0.883		29	50.6	18826	1.5
** > 1.5/d—6/d**	464	0.75	0.44 – 1.28	0.299	0.53	1.12	0.63 – 1.98	0.705	0.42	1.10	0.62 – 1.96	0.740	0.46	24	51.7	15228	1.6

^*^Asterisks indicate statistical significance

~ indicates results approached statistical significance

^a^beverages that were included in model 3 analyses

^b^CVD—was defined as the first event of definite or probable (fatal/non-fatal) coronary heart disease (CHD); including myocardial infarction (MI), angina pectoris, and death due to coronary heart disease (CHD), stroke, transient ischemic attack, heart failure, or peripheral artery disease from 1985 (mean age 24.5 y) through approximately 35 years of follow-up

^c^CARDIA Study—Coronary Artery Risk Development in Young Adults Study

^d^HFCS – high fructose corn syrup which contains more excess-free-fructose and higher fructose-to-glucose ratios than safe, as measured by independent labs, i.e., 5 – 9 of unpaired / excess-free-fructose g / 250 ml [20, 60, 61]

^e^Age – a 2-level variable defined as 18–24 and 25–30 y

^f^Low density lipoprotein cholesterol (LDL-C) serum concentration – a continuous variable

^g^Serum triglyceride concentration (a continuous variable)

^h^Serum apolipoprotein B concentration (a continuous variable)

^i^Orange juice—a low excess-free-fructose juice (0.4 g / 250 ml)

## Discussion

HFCS sweetened beverage intake was associated with higher CVD risk/incidence, independent of sex, race, education, weight, smoking, physical activity, dietary factors, and health indicators at enrollment. CVD risks were higher than associated with smoking. CVD risks rose from 12 to 71% with increasing intake. There were no associations with 100% orange juice intake. This is consistent with prior research [4, 5] and remarkable because, from a total sugars and total fructose perspective, these beverages are comparable. Cola contains 26 g of total sugars [60–62] and 16–17 g of total fructose / 250 ml [20], which is *not* materially different than orange juice with 21 g of total sugars and 11 g of total fructose / 250 ml [62]. What differs is their excess-free-fructose (EFF) content which is 5 – 9 g in cola vs. 0.4 g in orange juice / 250 ml [20, 60–62]. High EFF is problematic because 10 g triggers fructose malabsorption in adults [49–59].

HFCS sweetened beverage intake was associated with higher CVD risk/incidence among Black participants only. CVD *risk* was more than 2 times higher at ≥ 3 times/wk consumption, relative to ≤ 2 times/wk. The probability of an ordered relationship approached significance. CVD *incidence* was ~ 64% higher among multiple times/d Black HFCS sweetened beverage consumers vs. ≤ 2 times/wk. CVD risks were 12% higher with each cup consumed. Among White participants, associations *were not* significant across intake quartiles. However, there was a significant 14% increase in CVD risk with each cup consumed when expressed linearly. CVD risks among Black participants are likely understated given the disproportionately higher loss-to-follow-up among Black *daily* HFCS sweetened beverage consumers. CVD incidence was 45% higher among Black ≤ 2 times/wk HFCS consumers than 2 times/wk to once/day White consumers. CVD incidence was 136% higher among Black > 2 to 6 times/wk consumers, than among White consumers at comparable intake. These results are consistent with the hypothesis that CVD risk/incidence is *higher at lower* HFCS sweetened beverage intake among Black individuals.

CVD risk factors [23–26, 32, 40, 65–73, 83–88], indicators of kidney injury and gut dysbiosis, including hyperuricemia [33–35], hyperinsulinemia [24, 25, 70], hypercholesterolemia [24, 25, 44], high-risk serum apolipoprotein B/A1 ratios [44], and pre-diabetes [42, 45] *increased stepwise,* with *increasing* intake of HFCS sweetened beverages, among Black and White participants, but not hypertriglyceridemia. Among Black participants, hypertriglyceridemia remained *low* and increased nominally with increasing HFCS sweetened beverage intake, whereas it *increased significantly with increasing* HFCS sweetened beverage intake, among White participants. Unpaired fructose that *is* absorbed and metabolized significantly *increases* postprandial TG [89–91]. Differences in Black/White serum TG are consistent with higher fructose malabsorption among Black individuals [4, 14, 15]. Serum TG remained flat across orange juice intake levels, among all participants, which is consistent with research by Tappy et al., which showed that *paired* fructose / glucose slows unchecked fructose metabolism / catabolism and, thereby, its end-products [92].

Hyperuricemia increased with increasing HFCS sweetened beverage consumption, but at much higher rates and concentrations among White than Black participants. Uric acid increases parallelled increases in serum TG, among White participants, which is consistent with unchecked fructose metabolism. Unpaired fructose that *is* absorbed and metabolized *increases* serum uric acid [89–91]. Among Black participants, serum uric acid concentrations were lower despite more frequent HFCS sweetened beverage intake, which is also consistent with higher fructose malabsorption among Black individuals. Unpaired fructose is known to trigger gut dysbiosis, an overlooked source of serum uric acid [21, 25, 33–38, 49, 50]. High serum uric acid concentration is an accurate predictor of mortality *after* acute myocardial infarction (MI) [90]. Among Black participants, hyperuricemia *decreased* with *increasing* orange juice intake. This finding is also consistent with research by Tappy et al. (2016), which explored the protective effects of co-ingestion of glucose and fructose (paired fructose [92]). Since then, the Nestlé Company removed HFCS from many of its products [93].

Beisner et.al., [71] also found that high doses of [unpaired] fructose saturate the capacity to absorb (spill-over to the gut) and to catabolize fructose (spill-over to the liver). Differences in “high fructose syrup” absorption capacity, drive differences in “spill-over” effects. Higher absorption capacity leads to more “spill over” to the liver, higher serum lipids, uric acid accumulations, and steatosis, as observed among White participants. More “spill over” to the intestines/colon (lower absorption) leads to *lower* serum TG and uric acid accumulation, but a more altered gut microbiome [71]. Which may be occurring among Black participants. Orange juice contributes less to both forms of “spill over,” due to its 1:1 fructose-to-glucose ratio.

Hypertension *increased* with *increasing* HFCS sweetened beverage consumption, among Black participants. Conversely, it was *lowest* among White *daily* HFCS consumers. Uric acid is a hypertension risk factor [33–38]; however, it appears to be driving increases in hypertension among Black participants only. This paradox may be due to a lack of short chain fatty acids (SCFA) among Black individuals—a consequence of gut dysbiosis [21–38]. An out of balance gut microbiome promotes kidney injury, hypertension, and heart disease not only by *increasing* the number of uremic-toxin-producing-bacteria, it also *lowers the number of SCFA producing bacteria* [21–38, 94–97]. Lower SCFAs foster CVD because SCFAs improve gut barrier integrity, glucose and lipid metabolism, help regulate the immune system and the inflammatory response, and are instrumental in *lowering* blood pressure. Low gut bacterial diversity and composition are predictive of hypertension [95], and elevated blood pressure was transferrable through microbiota [27]. Recent research shows that even moderate HFCS sweetened beverage intake is associated with elevated serum sodium – a hypertension risk factor [98]. Low SCFAs may also explain the fact that Black ≤ 2 times/wk HFCS sweetened beverage consumers had significantly *higher* hyperinsulinemia (7.3%) than White *multiple times/d* consumers (4.0%), because low SCFAs is associated with *lower* insulin clearance and higher odds of dysglycemia [99, 100]. Reduced insulin clearance, among Black participants, may also be compensatory [99, 100]. It may reflect diminished biological effectiveness of GIP due to in situ GIP fructosylation via the Maillard reaction which has been shown to diminish its biological effectiveness and result in insulin *insufficiency* [68].

Among White participants, the rate of increase of ApoB to A1 ratios was significantly *higher* with increasing HFCS sweetened beverage than with orange juice, which is consistent with unchecked unpaired fructose-metabolism-driven increases in TG and thereby ApoB. Apolipoprotein B (ApoB) is the major structural protein of chylomicrons. ApoB concentrations increase to carry TG throughout the body. ApoB is also the major protein of atherogenic lipoproteins (VLDL, IDL, LDL, remnant Cholesterol (RC)) – the “bad” atherosclerotic cholesterol. These particles have one molecule of ApoB. Increases in ApoB concentration are also a consequence of gut dysbiosis to clear toxic LPS which rise due to gut bacterial LPS overproduction [71, 72]. ApoB serve a clearance function but are also atherosclerotic [38–45]. ApoA lipoproteins carry HDL – the "good" cholesterol. Apolipoprotein B to A1 ratios are strong predictors of heart disease [101]. However, ApoB to A1 ratios appear less predictive of heart disease in Black individuals, as ratios *increased* uniformly *across beverages*, including orange juice, *which was not* associated with CVD. However, gut dysbiosis can impair cholesterol elimination and contribute to the progression of atherosclerotic plaque [102]. Scripps researchers found they could reduce plasma total cholesterol concentration and atherosclerotic plaques when they selectively remodeled and rebalanced the gut microbiome [103].

The serum concentration of advanced glycation end-products (AGE) and the ratio of AGE to soluble RAGE – the receptor isoform that quenches RAGE signaling [69] may be overlooked CVD risk factors among Black individuals. Brinkley et. al. found that the Carboxymethyllysine (CML) to sRAGE ratio is high in Black individuals as compared to White individuals. CML is a well-studied AGE [83, 84]. They hypothesized that the ratio is high because Black individuals have higher AGE burden than White individuals [69]. This is plausibly explained by higher fructose malabsorption among Black individuals and high unpaired fructose reactivity in the gut which is high because fructose is in open chain form 400 times more than glucose [48, 83]. The pH in the duodenum promotes its reactivity [83, 104], and the phosphates in soda (particularly cola) catalyze the Maillard reaction [104]. AGE in circulation trigger vascular smooth muscle cells to produce excessive extracellular matrix proteins, which contribute to stenosis and atherosclerosis [105]. AGE in the gut disrupt the microbiome [106], and when absorbed are atherosclerotic, and proinflammatory [69, 105, 106]. High AGE burden / RAGE signaling, as measured by skin autofluorescence, is associated with increased risk of CVD mortality [73, 107]. The high ratio of AGE to sRAGE has been independently associated with albuminuria, a marker of kidney disease, in patients with hypertension [108].

Results herein resemble research with nationally representative data. Consumers of HFCS sweetened beverages and apple juice 5 or more times/wk were nearly 3 times more likely to have coronary heart disease (CHD) than less frequent/never consumers, and orange juice intake appeared protective [5]. Our findings also resemble Jackson Heart Study (JHS) results of older Black individuals. CHD risk was ~ *2.6 times* higher with *daily* HFCS sweetened beverage consumption vs. less frequent/never, independent of confounders [4]. In the JHS, 85% of participants had *normal* fasting TG concentration even though ~ 60% consumed HFCS sweetened beverages ≥ once/d [4]. At enrollment, only 3% of Black CARDIA participants had hypertriglyceridemia, even though 56.2%% consumed HFCS sweetened beverages ≥ once/d. In the California Teachers Study (CTS), wherein most participants were non-Hispanic White women (*n* = 106,178) [1], CVD risks were ~ *1.4 times* higher among daily HFCS sweetened beverage consumers vs. seldom/ never, i.e., risks that are much *lower* than found herein, among Black participants, and in the JHS. Fructose malabsorption prevalence differences may contribute to these disparate CVD risks.

Substituting HFCS sweetened beverages with sucrose sweetened coffee and tea *reduced* CHD risk/incidence in another US study [109]. Researchers hypothesized that something in coffee and tea may account for the lower CHD risk. An alternative explanation is that the lower risk lies in the fructose-to-glucose ratio differences between HFCS and sucrose. Sucrose, a disaccharide of fructose and glucose, does not trigger fructose malabsorption [57], except in young children [52, 53, 57]. In Japan, there was no increase in CHD (ischemic heart disease) risk, among regular soda drinkers [110]. What differs between Japan and the US is the HFCS which, in Japan, is limited by government statute. Conversely, several largescale US studies showed significant associations between HFCS sweetened beverages and CHD/CVD risk/incidence and mortality [1–6], independent of potential confounders.

In another study, there was a significant dose-dependent relationship between “added sugars” intake and CVD mortality with nationally representative US study data (NHANES) [111]. Added sugars included all sugars used in processed or prepared foods (HFCS and sucrose), but not naturally occurring sugar, as in fruits and fruit juices. The time period of the study (1988–2010) coincides with peak HFCS consumption (approximately 80 g p/d / about ~ 1 lb/wk, 1999), as reported before retroactively-applied subjective increases in consumer-level loss allowances [20, 112–114]. When stratified by race, the association was significant among non-Hispanic Whites only [111]. One possible explanation for this finding is exclusion bias due to pre-existing heart disease, T2D, and missing dietary data that *were not* analyzed by race. Non-Hispanic Black participants may have been disproportionately excluded from the analysis, as heart disease develops at a younger age in Black individuals [115].

Our findings are consistent with the TG paradox in individuals of African descent [14]. Even though insulin resistance, CVD, and T2D are associated with hypertriglyceridemia, Black individuals with these conditions usually have *normal* TG. While higher activity of the enzyme that clears TG rich lipid particles is plausible, it is reasonable to suggest, given our results and those from other studies, that higher fructose malabsorption prevalence and gut resident mechanisms more plausibly explain the link between HFCS sweetened beverage intake and CVD in Black individuals. This is consistent with emerging gut/heart axis research [21–45] and with the fact that the Black/White death disparity began increasing in the US in the early 1980’s [12, 13], coincident with the shift from sucrose to HFCS in US soft drinks (~ 1980–1984) [16–19, 46, 47, 113, 114], and its proliferation in the US food supply [20, 77, 111–114, 116–118]. Since then, Black adults have had consistently worse cardiovascular health than non-Hispanic White adults.

The CVD role of high unpaired fructose sweeteners has received less attention than fats, in part, due to researchers disclosed [119] and undisclosed conflicts of interests [120], and industry messaging that HFCS is “just like sugar” [121, 122]. This messaging ended after an undisclosed settlement agreement between US corn refiners and the US Sugar Association. However, misperceptions persist, and likely contribute to the lack of research momentum. Average per capita HFCS intake peaked in 1999, at approximately 80 g/d (~ 1 lb/wk), 14 years after the start of the CARDIA Study, and average per capita excess-free-fructose dosages, from HFCS, began exceeding levels that trigger fructose malabsorption (5—10 g) in the early 1980’s, i.e., shortly before the start of the CARDIA study (1985–1986) [20]. The unpaired fructose in one can of cola with 65% fructose/ 35% glucose is 12 g. By the end of the 35-y follow-up period, CARDIA participants had been exposed to higher levels of excess-free-fructose than generations before them.

In 2015, industry sponsored research aimed to measure the fructose-to-glucose ratio in the HFCS in popular sodas. They utilized different technologies which identified a low concentration of maltose and small chain glucose oligomers that were undetected by independent labs [123]. However, their findings are not relevant in the context of fructose malabsorption. There is no evidence, that we know of, wherein glucose polymers improve unpaired fructose/excess-free-fructose absorption. Co-ingestion of glucose did *not* improve fructose absorption or symptoms when applied to whole food containing fructose in excess of glucose [124]. Differences in the carbohydrate response element binding protein (ChREBP) gene may underlie differences in unpaired fructose absorption capacity across individuals [49, 51].

## Conclusion

The ubiquitous presence of HFCS, in the US food supply over the past 40 years, at higher fructose-to-glucose ratios than generally-recognized-as-safe, may have contributed to racial disparities in CVD/CVD mortality, due to the following reasons: higher fructose malabsorption prevalence among Black individuals, relative to others; high/unsafe unpaired fructose in the HFCS in beverages; high/unsafe fructose-to-glucose ratios in HFCS (1.5:1 – 1.9:1); high unabsorbed unpaired fructose induced gut dysbiosis and its health consequences (hypertension, kidney injury, elevated atherogenic LPS and proinflammatory uric acid, reduced cholesterol clearance, diminished short chain fatty acid (SCFA) protection, and gut hormone dysregulation); and high unabsorbed unpaired fructose induced gut reactivity and its health consequences (formation of pro-inflammatory atherosclerotic FruAGE, and dysregulation of incretins (GIP and GLP-1) which leads to weight gain, insulin *insufficiency* and hyperglycemia). Fructose malabsorption may be an overlooked CVD risk factor, and more attention to CVD consequences of HFCS is needed, including further investigation of fructose malabsorption. More comprehensive nutrition facts, food warning labels, and better food safety oversight should also be considered.

## Limitations

This study has limitations. It may not be generalizable, as the CARDIA study is specific to young adults living in specific regions. However, results are consistent with findings of Black individuals in the JHS [4], and with other studies of HFCS sweetened beverages and heart disease [1–3, 5–7]. Second, results are based on a combination of inputs that included self-reports, which may be subject to reporting bias. However, associations are consistent with existing literature [1–7]. Third, HFCS is not exclusive to beverages. One third of all HFCS consumed in the US is from food [16–19, 117, 118], and there are other sources of unpaired-free-fructose (agave syrup [63], crystalline fructose, apple juice/powder)/ (apples, pears, watermelon, and mangoes) [62] that contribute to daily unpaired free-fructose-dosages/load. Therefore, we may be underestimating the CVD risk from HFCS. Fourth, loss-to-follow-up was disproportionately higher among Black participants, particularly among daily HFCS consumers, which may have contributed to bias and underestimation of CVD risk/incidence at higher intakes. Fifth, we were not able to control for measures/types of visceral fat, as they were available only for a small subset of participants. It is thought that both the total amount of body fat and the *location* of excess body fat contribute to CVD risk [125].

## Data Availability

Not applicable.

## Abbreviations

AGE: Advanced Glycation End-products
BMI: Body Mass Index
BP: Blood Pressure
CARDIA: Coronary Artery Risk Development in Young Adults Study
CDC: United States Centers for Disease Control
CHD: Coronary Heart Disease
CI: Confidence Interval
CVD: Cardiovascular Disease
DNL: De novo lipogenesis
EFF: Excess-free-fructose
enFruAGE: Enteric fructose-formed advanced glycation end-products
BaGLP-1: Glucagon-like Peptide-1
GIP: Gastric Inhibitory Polypeptide
GRAS: Generally Recognized as Safe
HFCS: High Fructose Corn Syrup
HR: Hazard Ratio
JHS: The Jackson Heart Study
LDL-C: Low Density Lipoprotein
MI: Myocardial Infarction
NHANES: US National Health and Nutrition Examination Survey
RAGE: Receptor of Advanced Glycation End-products
SSB: Sugar Sweetened Beverages
T2D: Type 2 Diabetes
TG: Triglycerides
ttlEFF: Any combination of beverages high in excess-free-fructose including high fructose corn syrup sweetened soda, fruit drinks, and apple juice
uACR: Urinary albumin to creatinine ratio, a marker of kidney injury/ disease
US: United States
VLDL: Very Low-Density Lipoprotein

## References

[1] Pacheco LS, Lacey JV Jr, Martinez ME, Lemus H, Araneta MRG, Sears DD, Talavera GA, Anderson CAM. Sugar-sweetened beverage intake and cardiovascular disease risk in the California teachers study. J Am Heart Assoc. 2020;9(10):e014883. 10.1161/JAHA.119.014883. https://pubmed.ncbi.nlm.nih.gov/32397792/. Epub 2020 May 13. PMID: 32397792; PMCID: PMC7660873. 32397792 10.1161/JAHA.119.014883 PMC7660873

[2] de Koning L, Malik VS, Kellogg MD, Rimm EB, Willett WC, Hu FB. Sweetened beverage consumption, incident coronary heart disease, and biomarkers of risk in men. Circulation. 2012;125(14):1735–41, S1. 10.1161/CIRCULATIONAHA.111.067017. https://www.ncbi.nlm.nih.gov/pubmed/22412070. Epub 2012 Mar 12. PubMed PMID: 22412070; PubMed Central PMCID: PMC3368965. 22412070 10.1161/CIRCULATIONAHA.111.067017 PMC3368965

[3] Fung TT, Malik V, Rexrode KM, Manson JE, Willett WC, Hu FB. Sweetened beverage consumption and risk of coronary heart disease in women. Am J Clin Nutr. 2009;89(4):1037–42. 10.3945/ajcn.2008.27140. https://www.ncbi.nlm.nih.gov/pubmed/19211821. 19211821 10.3945/ajcn.2008.27140 PMC2667454

[4] DeChristopher LR, Auerbach BJ, Tucker KL. High fructose corn syrup, excess-free-fructose, and risk of coronary heart disease among African Americans– the Jackson Heart Study. BMC Nutr. 2020;6:70. 10.1186/s40795-020-00396-x. https://pubmed.ncbi.nlm.nih.gov/33292663/. 33292663 10.1186/s40795-020-00396-x PMC7722296

[5] DeChristopher LR, Uribarri J, Tucker KL. Intake of high-fructose corn syrup sweetened soft drinks, fruit drinks and apple juice is associated with prevalent coronary heart disease in US adults, aged 20–30 years. BMC Nutr J. 2017;3:51. 10.1186/s40795-017-0168-9. https://bmcnutr.biomedcentral.com/articles/10.1186/s40795-017-0168-9. 10.1186/s40795-017-0168-9 PMC7050890 32153831

[6] Yin J, Zhu Y, Malik V, Li X, Peng X, Zhang FF, Shan Z, Liu L. Intake of sugar-sweetened and low-calorie sweetened beverages and risk of cardiovascular disease: a meta-analysis and systematic review. Adv Nutr. 2021;12(1):89–101. 10.1093/advances/nmaa084. 32696948 10.1093/advances/nmaa084 PMC7850046

[7] Narain A, Kwok CS, Mamas MA. Soft drinks and sweetened beverages and the risk of cardiovascular disease and mortality: a systematic review and meta-analysis. Int J Clin Pract. 2016;70(10):791–805. https://pubmed.ncbi.nlm.nih.gov/27456347/. 27456347 10.1111/ijcp.12841

[8] National Center for Health Statistics. Health, United States, 2017. Table 19: Leading causes of death and numbers of deaths, by sex, race, and Hispanic origin: United States, 1980 and 2016. Hyattsville: National Center for Health Statistics; 2018. https://www.cdc.gov/nchs/data/hus/2017/019.pdf. Accessed 23 Jan 2023

[9] Heart Disease Facts. Centers for disease control. https://www.cdc.gov/heart-disease/data-research/facts-stats/?CDC_AAref_Val=https://www.cdc.gov/heartdisease/facts.htm. Accessed 12 July 2024.

[10] Gu T, Chu Q, Yu Z, Fa B, Li A, Xu L, Wu R, He Y. History of coronary heart disease increased the mortality rate of patients with COVID-19: a nested case-control study. BMJ Open. 2020;10(9):e038976. 10.1136/bmjopen-2020-038976. https://pubmed.ncbi.nlm.nih.gov/32948572/. PMID: 32948572; PMCID: PMC7499679. 32948572 10.1136/bmjopen-2020-038976 PMC7499679

[11] Malik VS, Hu FB. Sugar-sweetened beverages and cardiometabolic health: an update of the evidence. Nutrients. 2019;11(8):1840. 10.3390/nu11081840 . https://pubmed.ncbi.nlm.nih.gov/31398911/. PMID: 31398911; PMCID: PMC6723421. 31398911 10.3390/nu11081840 PMC6723421

[12] Kramer MR, Valderrama AL, Casper ML. Decomposing black-white disparities in heart disease mortality in the United States, 1973–2010: an age-period-cohort analysis. Am J Epidemiol. 2015;182(4):302–12. 10.1093/aje/kwv050. https://www.ncbi.nlm.nih.gov/pubmed/26199382. 26199382 10.1093/aje/kwv050 PMC4528952

[13] United States Department of Health and Human Services Office of Minority Health. Heart Disease and African Americans. https://minorityhealth.hhs.gov/omh/browse.aspx?lvl=4&lvlid=19. Accessed 1 Oct 2023.

[14] Yu SS, Castillo DC, Courville AB, Sumner AE. The triglyceride paradox in people of African descent. Metab Syndr Relat Disord. 2012;10(2):77–82. 10.1089/met.2011.0108. https://www.ncbi.nlm.nih.gov/pmc/articles/PMC3311911/. 22224930 10.1089/met.2011.0108 PMC3311911

[15] Bacha F, Saad R, Gungor N, Janosky J, Arslanian SA. Obesity, regional fat distribution, and syndrome X in obese black versus white adolescents: race differential in diabetogenic and atherogenic risk factors. J Clin Endocrinol Metab. 2003;88(6):2534–40. https://pubmed.ncbi.nlm.nih.gov/12788850/. PubMed PMID: 12788850. 12788850 10.1210/jc.2002-021267

[16] U.S. Department of Agriculture, Economic Research Service. Sugars and sweeteners, background, high fructose corn syrup production and prices. http://www.ers.usda.gov/topics/crops/sugar-sweeteners/background/.

[17] Duffey KJ, Popkin BM. High-fructose corn syrup: is this what’s for dinner? Am J Clin Nutr. 2008;88(6):1722S-1732S. 10.3945/ajcn.2008.25825C. https://pubmed.ncbi.nlm.nih.gov/19064537/. PubMed PMID: 19064537; PubMed Central PMCID: PMC2746720. 19064537 10.3945/ajcn.2008.25825C PMC2746720

[18] Morrison RM, Buzby JC. Guess who’s turning 100? Tracking a century of American eating. United States Department of Agriculture, Economic Research Service. Amber Waves. 2010. https://www.ers.usda.gov/amber-waves/2010/march/guess-who-s-turning-100tracking-a-century-of-american-eating/. Accessed 22 Dec 2017.

[19] Wells HF, Buzby JC. High-fructose corn syrup usage may be leveling off. United States Department of Agriculture. Economic Research Service. 2008. https://www.ers.usda.gov/amber-waves/2008/february/high-fructose-corn-syrup-usage-may-be-leveling-off/. Accessed 22 Dec 2017.

[20] DeChristopher LR. 40 years of adding more fructose to high fructose corn syrup than is safe, through the lens of malabsorption and altered gut health–gateways to chronic disease. Nutr J. 2024;23:16. 10.1186/s12937-024-00919-3. 38302919 10.1186/s12937-024-00919-3 PMC10835987

[21] Cheng WL, Li SJ, Lee TI, Lee TW, Chung CC, Kao YH, Chen YJ. Sugar fructose triggers gut dysbiosis and metabolic inflammation with cardiac arrhythmogenesis. Biomedicines. 2021;9(7):728. 10.3390/biomedicines9070728. https://pubmed.ncbi.nlm.nih.gov/34201938/. PMID: 34201938; PMCID: PMC8301417. 34201938 10.3390/biomedicines9070728 PMC8301417

[22] Ascher S, Reinhardt C. The gut microbiota: an emerging risk factor for cardiovascular and cerebrovascular disease. Eur J Immunol. 2018;48(4):564–75. 10.1002/eji.201646879. Epub 2018 Jan 19 PMID: 29230812. 29230812 10.1002/eji.201646879

[23] Kazemian N, Mahmoudi M, Halperin F, et al. Gut microbiota and cardiovascular disease: opportunities and challenges. Microbiome. 2020;8:36. 10.1186/s40168-020-00821-0. https://microbiomejournal.biomedcentral.com/articles/10.1186/s40168-020-00821-0. 32169105 10.1186/s40168-020-00821-0 PMC7071638

[24] Witkowski M, Weeks TL, Hazen SL. Gut microbiota and cardiovascular disease. Circ Res. 2020;127(4):553–70. 10.1161/CIRCRESAHA.120.316242. https://pubmed.ncbi.nlm.nih.gov/32762536/. Epub 2020 Jul 30. PMID: 32762536; PMCID: PMC7416843. 32762536 10.1161/CIRCRESAHA.120.316242 PMC7416843

[25] Hsu CN, Yu HR, Chan JYH, Wu KLH, Lee WC, Tain YL. The impact of gut microbiome on maternal fructose intake-induced developmental programming of adult disease. Nutrients. 2022;14(5):1031. 10.3390/nu14051031. https://pubmed.ncbi.nlm.nih.gov/35268005/. PMID: 35268005; PMCID: PMC8912426. 35268005 10.3390/nu14051031 PMC8912426

[26] Novakovic M, Rout A, Kingsley T, Kirchoff R, Singh A, Verma V, Kant R, Chaudhary R. Role of gut microbiota in cardiovascular diseases. World J Cardiol. 2020;12(4):110–22. 10.4330/wjc.v12.i4.110. https://www.ncbi.nlm.nih.gov/pmc/articles/PMC7215967/. PMID: 32431782; PMCID: PMC7215967. 32431782 10.4330/wjc.v12.i4.110 PMC7215967

[27] Li J, Zhao F, Wang Y, Chen J, Tao J, Tian G, Wu S, Liu W, Cui Q, Geng B, Zhang W, Weldon R, Auguste K, Yang L, Liu X, Chen L, Yang X, Zhu B, Cai J. Gut microbiota dysbiosis contributes to the development of hypertension. Microbiome. 2017;5(1):14. 10.1186/s40168-016-0222-x. https://microbiomejournal.biomedcentral.com/articles/10.1186/s40168-016-0222-x. PMID: 28143587; PMCID: PMC5286796. 28143587 10.1186/s40168-016-0222-x PMC5286796

[28] Sun S, Lulla A, Sioda M, Winglee K, Wu MC, Jacobs DR Jr, Shikany JM, Lloyd-Jones DM, Launer LJ, Fodor AA, Meyer KA. Gut microbiota composition and blood pressure. Hypertension. 2019;73(5):998–1006. 10.1161/HYPERTENSIONAHA.118.12109. https://pubmed.ncbi.nlm.nih.gov/30905192/. PMID: 30905192; PMCID: PMC6458072. 30905192 10.1161/HYPERTENSIONAHA.118.12109 PMC6458072

[29] Pevsner-Fischer M, Blacher E, Tatirovsky E, Ben-Dov IZ, Elinav E. The gut microbiome and hypertension. Curr Opin Nephrol Hypertens. 2017;26(1):1–8. 10.1097/MNH.0000000000000293. https://pubmed.ncbi.nlm.nih.gov/27798455/. PMID: 27798455. 27798455 10.1097/MNH.0000000000000293

[30] Louca P, Nogal A, Wells PM, Asnicar F, Wolf J, Steves CJ, Spector TD, Segata N, Berry SE, Valdes AM, Menni C. Gut microbiome diversity and composition is associated with hypertension in women. J Hypertens. 2021;39(9):1810–6. 10.1097/HJH.0000000000002878. https://pubmed.ncbi.nlm.nih.gov/33973959/. PMID: 33973959; PMCID: PMC7611529. 33973959 10.1097/HJH.0000000000002878 PMC7611529

[31] Carding S, Verbeke K, Vipond DT, Corfe BM, Owen LJ. Dysbiosis of the gut microbiota in disease. Microb Ecol Health Dis. 2015;26:26191. 10.3402/mehd.v26.26191. https://www.ncbi.nlm.nih.gov/pmc/articles/PMC4315779/. Published 2015 Feb 2. 25651997 10.3402/mehd.v26.26191 PMC4315779

[32] Sanchez-Rodriguez E, Egea-Zorrilla A, Plaza-Díaz J, et al. The gut microbiota and its implication in the development of atherosclerosis and related cardiovascular diseases. Nutrients. 2020;12(3):605. 10.3390/nu12030605. https://www.ncbi.nlm.nih.gov/pmc/articles/PMC7146472/. Published 2020 Feb 26. 32110880 10.3390/nu12030605 PMC7146472

[33] Chu Y, Sun S, Huang Y, Gao Q, Xie X, Wang P, Li J, Liang L, He X, Jiang Y, Wang M, Yang J, Chen X, Zhou C, Zhao Y, Ding F, Zhang Y, Wu X, Bai X, Wu J, Wei X, Chen X, Yue Z, Fang X, Huang Q, Wang Z, Huang R. Metagenomic analysis revealed the potential role of gut microbiome in gout. NPJ Biofilms Microbiomes. 2021;7(1):66. 10.1038/s41522-021-00235-2. https://www.nature.com/articles/s41522-021-00235-2. PMID: 34373464; PMCID: PMC8352958. 34373464 10.1038/s41522-021-00235-2 PMC8352958

[34] Shao T, Shao L, Li H, Xie Z, He Z, Wen C. Combined signature of the fecal microbiome and metabolome in patients with gout. Front Microbiol. 2017;8:268. 10.3389/fmicb.2017.00268. https://pubmed.ncbi.nlm.nih.gov/28270806/. Published 2017 Feb 21. 28270806 10.3389/fmicb.2017.00268 PMC5318445

[35] Wang Z, Li Y, Liao W, Huang J, Liu Y, Li Z, Tang J. Gut microbiota remodeling: a promising therapeutic strategy to confront hyperuricemia and gout. Front Cell Infect Microbiol. 2022;12:935723. 10.3389/fcimb.2022.935723. https://www.ncbi.nlm.nih.gov/pmc/articles/PMC9399429/. PMID: 36034697; PMCID: PMC9399429. 36034697 10.3389/fcimb.2022.935723 PMC9399429

[36] Cao C, Zhu H, Yao Y, Zeng R. Gut dysbiosis and kidney diseases. Front Med (Lausanne). 2022;9:829349. 10.3389/fmed.2022.829349. https://www.ncbi.nlm.nih.gov/pmc/articles/PMC8927813/. PMID: 35308555; PMCID: PMC8927813. 35308555 10.3389/fmed.2022.829349 PMC8927813

[37] Johnson RJ, Sanchez-Lozada LG, Nakagawa T. The effect of fructose on renal biology and disease. J Am Soc Nephrol. 2010;21(12):2036–9. 10.1681/ASN.2010050506. https://pubmed.ncbi.nlm.nih.gov/21115612/. Epub 2010 Nov 29. PMID: 21115612. 21115612 10.1681/ASN.2010050506

[38] Tong S, Zhang P, Cheng Q, Chen M, Chen X, Wang Z, Lu X, Wu H. The role of gut microbiota in gout: Is gut microbiota a potential target for gout treatment. Front Cell Infect Microbiol. 2022;12:1051682. 10.3389/fcimb.2022.1051682. https://pubmed.ncbi.nlm.nih.gov/36506033/. PMID: 36506033; PMCID: PMC9730829. 36506033 10.3389/fcimb.2022.1051682 PMC9730829

[39] Le Roy T, Lécuyer E, Chassaing B, et al. The intestinal microbiota regulates host cholesterol homeostasis. BMC Biol. 2019;17:94. 10.1186/s12915-019-0715-8. 31775890 10.1186/s12915-019-0715-8 PMC6882370

[40] Tang WH, Kitai T, Hazen SL. Gut microbiota in cardiovascular health and disease. Circ Res. 2017;120(7):1183–96. 10.1161/CIRCRESAHA.117.309715. https://pubmed.ncbi.nlm.nih.gov/28360349/. PMID: 28360349; PMCID: PMC5390330. 28360349 10.1161/CIRCRESAHA.117.309715 PMC5390330

[41] Dargent A, Pais de Barros JP, Saheb S, Bittar R, Le Goff W, Carrié A, Gautier T, Fournel I, Rerole AL, Choubley H, Masson D, Lagrost L, Quenot JP. LDL apheresis as an alternate method for plasma LPS purification in healthy volunteers and dyslipidemic and septic patients. J Lipid Res. 2020;61(12):1776–83. 10.1194/jlr.RA120001132. https://www.ncbi.nlm.nih.gov/pmc/articles/PMC7707173/. Epub 2020 Oct 9. PMID: 33037132; PMCID: PMC7707173. 33037132 10.1194/jlr.RA120001132 PMC7707173

[42] Yu Y, Raka F, Adeli K. The role of the gut microbiota in lipid and lipoprotein metabolism. J Clin Med. 2019;8(12):2227. 10.3390/jcm8122227. https://www.ncbi.nlm.nih.gov/pmc/articles/PMC6947520/. PMID: 31861086; PMCID: PMC6947520. 31861086 10.3390/jcm8122227 PMC6947520

[43] Vreugdenhil AC, Rousseau CH, Hartung T, Greve JW, van ‘t Veer C, Buurman WA. Lipopolysaccharide (LPS)-binding protein mediates LPS detoxification by chylomicrons. J Immunol. 2003;170(3):1399–405. 10.4049/jimmunol.170.3.1399. https://pubmed.ncbi.nlm.nih.gov/12538700/. PMID: 12538700. 12538700 10.4049/jimmunol.170.3.1399

[44] Vreugdenhil AC, Snoek AM, van ‘t Veer C, Greve JW, Buurman WA. LPS-binding protein circulates in association with apoB-containing lipoproteins and enhances endotoxin-LDL/VLDL interaction. J Clin Invest. 2001;107(2):225–34. 10.1172/JCI10832. https://pubmed.ncbi.nlm.nih.gov/11160139/. PMID: 11160139; PMCID: PMC199173. 11160139 10.1172/JCI10832 PMC199173

[45] Gérard C, Vidal H. Impact of gut microbiota on host glycemic control. Front Endocrinol (Lausanne). 2019;10:29. 10.3389/fendo.2019.00029. https://www.ncbi.nlm.nih.gov/pmc/articles/PMC6363653/. Published 2019 Jan 30. 30761090 10.3389/fendo.2019.00029 PMC6363653

[46] Kammer A. Cornography: perverse incentives and the United States corn subsidy. 2011. http://nrs.harvard.edu/urn-3:HUL.InstRepos:8965640.

[47] Harvie A, Wise TA. Sweetening the pot implicit subsidies to corn sweeteners and the U.S. obesity epidemic. Global Development and Environment Institute Tufts University. GDAE Policy Brief No. 2009. https://sites.tufts.edu/gdae/files/2020/03/PB09-01SweeteningPotFeb09.pdf, https://www.yumpu.com/en/document/view/11847453/sweetening-the-pot-implicit-subsidies-to-corn-tufts-university.

[48] Clemens RA, Jones JM,Kern M, Lee SY, Mayhew EJ, Slavin JL, and Zivanovic S. Functionality of sugars in foods and health. Compr Rev Food Sci Food Saf. 2016. 10.1111/1541-4337.12194, https://onlinelibrary.wiley.com/doi/full/10.1111/1541-4337.12194. 10.1111/1541-4337.12194 33401825

[49] Ferraris RP, Choe JY, Patel CR. Intestinal absorption of fructose. Annu Rev Nutr. 2018;38:41–67. 10.1146/annurev-nutr-082117-051707. https://pubmed.ncbi.nlm.nih.gov/29751733/. 29751733 10.1146/annurev-nutr-082117-051707 PMC6457363

[50] Payne AN, Chassard C, Lacroix C. Gut microbial adaptation to dietary consumption of fructose, artificial sweeteners and sugar alcohols: implications for host-microbe interactions contributing to obesity. Obes Rev. 2012;13(9):799–809. 10.1111/j.1467-789X.2012.01009.x. https://pubmed.ncbi.nlm.nih.gov/22686435/. Epub 2012 Jun 11. PMID: 22686435. 22686435 10.1111/j.1467-789X.2012.01009.x

[51] Hannou SA, Haslam DE, McKeown NM, Herman MA. Fructose metabolism and metabolic disease. J Clin Invest. 2018;128(2):545–55. 10.1172/JCI96702. https://pubmed.ncbi.nlm.nih.gov/29388924/. Epub 2018 Feb 1. Review. PubMed PMID: 29388924; PubMed Central PMCID: PMC5785258. 29388924 10.1172/JCI96702 PMC5785258

[52] Jones HF, Burt E, Dowling K, Davidson G, Brooks DA, Butler RN. Effect of age on fructose malabsorption in children presenting with gastrointestinal symptoms. J Pediatr Gastroenterol Nutr. 2011;52(5):581–4. 10.1097/MPG.0b013e3181fd1315. https://www.ncbi.nlm.nih.gov/pubmed/21502829. PubMed PMID: 21502829. 21502829 10.1097/MPG.0b013e3181fd1315

[53] Smith MM, Davis M, Chasalow FI, Lifshitz F. Carbohydrate absorption from fruit juice in young children. Pediatrics. 1995;95(3):340–4. https://www.ncbi.nlm.nih.gov/pubmed/7862470. PubMed PMID: 7862470. 7862470 10.1542/peds.95.3.340

[54] Riby JE, Fujisawa T, Kretchmer N. Fructose absorption. Am J Clin Nutr. 1993;58(5 Suppl):748S-753S. https://pubmed.ncbi.nlm.nih.gov/8213606/. Review. PubMed PMID: 8213606. 8213606 10.1093/ajcn/58.5.748S

[55] Ebert K, Witt H. Fructose malabsorption. Mol Cell Pediatr. 2016;3:10. 10.1186/s40348-016-0035-9. https://www.ncbi.nlm.nih.gov/pubmed/26883354. PubMed PMCID: PMC4755956. 26883354 10.1186/s40348-016-0035-9 PMC4755956

[56] Biesiekierski JR. Fructose-induced symptoms beyond malabsorption in FGID. United European Gastroenterol J. 2014;2(1):10–3. 10.1177/20506406135109055. https://www.ncbi.nlm.nih.gov/pmc/articles/PMC4040804/. 24918003 10.1177/20506406135109055 PMC4040804

[57] Gibson PR, Newnham E, Barrett JS, Shepherd SJ, Muir JG. Review article: fructose malabsorption and the bigger picture. Aliment Pharmacol Ther. 2007;25:349–63. https://www.ncbi.nlm.nih.gov/pubmed/17217453. 17217453 10.1111/j.1365-2036.2006.03186.x

[58] Rumessen JJ. Fructose and related food carbohydrates sources, intake, absorption, and clinical implications. Scand J Gastroenterol. 1992;27:819–28. https://www.ncbi.nlm.nih.gov/pubmed/1439534. 1439534 10.3109/00365529209000148

[59] Beyer PL, Caviar EM, McCallum RW. Fructose intake at current levels in the United States may cause gastrointestinal distress in normal adults. J Am Diet Assoc. 2005;105:1559–66. https://www.ncbi.nlm.nih.gov/pubmed/16183355. 16183355 10.1016/j.jada.2005.07.002

[60] Ventura EE, Davis JN, Goran MI. Sugar content of popular sweetened beverages based on objective laboratory analysis: focus on fructose content. Obesity (Silver Spring). 2011;19(4):868–74. 10.1038/oby.2010.255. https://www.ncbi.nlm.nih.gov/pubmed/20948525. Epub 2010 Oct 14. PubMed PMID: 20948525. 20948525 10.1038/oby.2010.255

[61] Walker RW, Dumke KA, Goran MI. Fructose content in popular beverages made with and without high-fructose corn syrup. Nutrition. 2014;30(7–8):928–35. 10.1016/j.nut.2014.04.003. https://www.sciencedirect.com/science/article/pii/S0899900714001920. Epub 2014 Apr 18. PubMed PMID: 24985013. 24985013 10.1016/j.nut.2014.04.003

[62] U.S. Department of Agriculture, Agricultural Research Service. USDA National Nutrient Database for Standard Reference, Release 28. Nutrient Data Laboratory Home Page, https://www.ars.usda.gov/ARSUserFiles/80400525/Data/SR/SR28/reports/sr28fg09.pdf. Accessed 23 Aug 2017.

[63] Mellado-Mojica E, López MG. Identification, classification, and discrimination of agave syrups from natural sweeteners by infrared spectroscopy and HPAEC-PAD. Food Chem. 2015;167:349–57. 10.1016/j.foodchem.2014.06.111. https://www.ncbi.nlm.nih.gov/pubmed/25148997. Epub 2014 Jul 9. PubMed PMID: 25148997. 25148997 10.1016/j.foodchem.2014.06.111

[64] Walker RW, Lê KA, Davis J, Alderete TL, Cherry R, Lebel S, Goran MI. High rates of fructose malabsorption are associated with reduced liver fat in obese African Americans. J Am Coll Nutr. 2012;31(5):369–74. https://pubmed.ncbi.nlm.nih.gov/23529994/, https://goranlab.com/publications/. PubMed PMID: 23529994. 23529994 10.1080/07315724.2012.10720445

[65] Hanssen NM, Wouters K, Huijberts MS, Gijbels MJ, Sluimer JC, Scheijen JL, Heeneman S, Biessen EA, Daemen MJ, Brownlee M, de Kleijn DP, Stehouwer CD, Pasterkamp G, Schalkwijk CG. Higher levels of advanced glycation endproducts in human carotid atherosclerotic plaques are associated with a rupture-prone phenotype. Eur Heart J. 2014;35(17):1137–46. 10.1093/eurheartj/eht402. https://pubmed.ncbi.nlm.nih.gov/24126878/. Epub 2013 Oct 14. PMID: 24126878. 24126878 10.1093/eurheartj/eht402

[66] Fishman SL, Sonmez H, Basman C, et al. The role of advanced glycation end-products in the development of coronary artery disease in patients with and without diabetes mellitus: a review. Mol Med. 2018;24:59. 10.1186/s10020-018-0060-3. https://pubmed.ncbi.nlm.nih.gov/30470170/. 30470170 10.1186/s10020-018-0060-3 PMC6251169

[67] Yan SF, Ramasamy R, Naka Y, Schmidt AM. Glycation, inflammation, and RAGE: a scaffold for the macrovascular complications of diabetes and beyond. Circ Res. 2003;93(12):1159–69. 10.1161/01.RES.0000103862.26506.3D. https://pubmed.ncbi.nlm.nih.gov/14670831/. PMID: 14670831. 14670831 10.1161/01.RES.0000103862.26506.3D

[68] O'Harte FP, Gray AM, Flatt PR. Gastric inhibitory polypeptide and effects of glycation on glucose transport and metabolism in isolated mouse abdominal muscle. J Endocrinol. 1998;156(2):237–43. 10.1677/joe.0.1560237. https://pubmed.ncbi.nlm.nih.gov/9518868/. PMID: 9518868. 9518868 10.1677/joe.0.1560237

[69] Brinkley TE, Leng X, Nicklas BJ, Kritchevsky SB, Ding J, Kitzman DW, Hundley WG. Racial differences in circulating levels of the soluble receptor for advanced glycation endproducts in middle-aged and older adults. Metabolism. 2017;70:98–106. 10.1016/j.metabol.2017.02.008. https://pubmed.ncbi.nlm.nih.gov/28403949/. Epub 2017 Feb 10. PMID: 28403949; PMCID: PMC5396843. 28403949 10.1016/j.metabol.2017.02.008 PMC5396843

[70] Jeevanandam J, Paramasivam E, Saraswathi NT. Glycation restrains open-closed conformation of Insulin. Comput Biol Chem. 2023;102:107803. 10.1016/j.compbiolchem.2022.107803. https://pubmed.ncbi.nlm.nih.gov/36542957/. Epub 2022 Dec 16. PMID: 36542957. 36542957 10.1016/j.compbiolchem.2022.107803

[71] Beisner J, Gonzalez-Granda A, Basrai M, Damms-Machado A, Bischoff SC. Fructose-induced intestinal microbiota shift following two types of short-term high-fructose dietary phases. Nutrients. 2020;12(11):3444. 10.3390/nu12113444. https://pubmed.ncbi.nlm.nih.gov/33182700/. PMID: 33182700; PMCID: PMC7697676. 33182700 10.3390/nu12113444 PMC7697676

[72] Aschner M, Skalny AV, Gritsenko VA, Kartashova OL, Santamaria A, Rocha JBT, Spandidos DA, Zaitseva IP, Tsatsakis A, Tinkov AA. Role of gut microbiota in the modulation of the health effects of advanced glycation end-products (Review). Int J Mol Med. 2023;51(5):44. 10.3892/ijmm.2023.5247. Epub 2023 Apr 13. PMID: 37052251; PMCID: PMC10198061. Role of gut microbiota in the modulation of the health effects of advanced glycation end-products (Review) - PMC (nih.gov). 37052251 10.3892/ijmm.2023.5247 PMC10198061

[73] Sharifi-Zahabi E, Sharafabad FH, Abdollahzad H, Malekahmadi M, Rad NB. Circulating advanced glycation end products and their soluble receptors in relation to all-cause and cardiovascular mortality: a systematic review and meta-analysis of prospective observational studies. Adv Nutr. 2021;12(6):2157–71. 10.1093/advances/nmab072. PMID: 34139010; PMCID: PMC8634502. Circulating Advanced Glycation End Products and Their Soluble Receptors in Relation to All-Cause and Cardiovascular Mortality: A Systematic Review and Meta-analysis of Prospective Observational Studies - PubMed (nih.gov). 34139010 10.1093/advances/nmab072 PMC8634502

[74] Martínez-Azcona O, Moreno-Álvarez A, Seoane-Pillado T, Niño-Grueiro I, Ramiro-Comesaña A, Menéndez-Riera M, Pérez-Domínguez M, Solar-Boga A, Leis-Trabazo R. Fructose malabsorption in asymptomatic children and in patients with functional chronic abdominal pain: a prospective comparative study. Eur J Pediatr. 2019;178(9):1395–403. 10.1007/s00431-019-03418-4. https://pubmed.ncbi.nlm.nih.gov/31325029/. Epub 2019 Jul 19. PMID: 31325029. 31325029 10.1007/s00431-019-03418-4

[75] Federal Register Volume 61, Number 165. August 23, 1996. Rules and Regulations, pages 43447-43450. From the Federal Register Online via the Government Publishing Office [FR Doc No.: 9621482]. https://www.gpo.gov/fdsys/pkg/FR-1996-08-23/html/96-21482.htm%20 Accessed 1 Oct 2023.

[76] Hong YM. Atherosclerotic cardiovascular disease beginning in childhood. Korean Circ J. 2010;40(1):1–9. 10.4070/kcj.2010.40.1.1. https://www.ncbi.nlm.nih.gov/pmc/articles/PMC2812791/. 20111646 10.4070/kcj.2010.40.1.1 PMC2812791

[77] Atkinson FS, Foster-Powell K, Brand-Miller JC. International tables of glycemic index and glycemic load values: 2008. Diabetes Care. 2008;31(12):2281–3. 10.2337/dc08-1239. http://care.diabetesjournals.org/content/31/12/2281. 18835944 10.2337/dc08-1239 PMC2584181

[78] Coronary Artery Risk Development in Young Adults (CARDIA) Study. https://www.nhlbi.nih.gov/science/coronary-artery-risk-development-young-adults-study-cardia, https://www.cardia.dopm.uab.edu/study-information/nhlbi-data-repository-data/cardia-documentation/78-cardia-documentation.

[79] Ogden CL, Kit BK, Carroll MD, Park S. Consumption of sugar drinks in the United States, 2005–2008. NCHS Data Brief. 2011;71:1–8. https://pubmed.ncbi.nlm.nih.gov/22617020/. PMID: 22617020. 22617020

[80] Morikawa N, Bancks MP, Yano Y, Kuwabara M, Gaffo AL, Duprez DA, Gross MD, Jacobs DR Jr. Serum urate trajectory in young adulthood and incident cardiovascular disease events by middle age: CARDIA study. Hypertension. 2021;78(5):1211–8. 10.1161/HYPERTENSIONAHA.121.17555. https://pubmed.ncbi.nlm.nih.gov/34092118/. Epub 2021 Jun 7. PMID: 34092118; PMCID: PMC8516664. 34092118 10.1161/HYPERTENSIONAHA.121.17555 PMC8516664

[81] McDonald A, Van Horn L, Slattery M, Hilner J, Bragg C, Caan B, Jacobs D Jr, Liu K, Hubert H, Gernhofer N, Betz E, Havlik D. The CARDIA dietary history: development, implementation, and evaluation. J Am Diet Assoc. 1991;91(9):1104–12. https://pubmed.ncbi.nlm.nih.gov/1918764/. PMID: 1918764. 1918764 10.1016/S0002-8223(21)01299-2

[82] Jacobs DR Jr, Hahn LP, Haskell WL, Pirie P, Sidney S. Validity and reliability of short physical activity history: cardia and the Minnesota heart health program. J Cardiopulm Rehabil. 1989;9(11):448–59. 10.1097/00008483-198911000-00003. https://pubmed.ncbi.nlm.nih.gov/29657358/. 29657358 10.1097/00008483-198911000-00003 PMC5894828

[83] DeChristopher LR. Consumption of Fructose and High Fructose Corn Syrup: Is Fructositis triggered bronchitis, asthma, & auto-immune reactivity merely a side bar in the Etiology of Metabolic Syndrome II (to be defined)? – Evidence and a Hypothesis. New York Medical College Library. 2012. Online at https://www.researchgate.net/publication/276920662_Consumption_of_Fructose_and_High_Fructose_Corn_Syrup_Is_Fructositis_triggered_bronchitis_asthma_auto-immune_reactivity_merely_a_side_bar_in_the_Etiology_of_Metabolic_Syndrome_II_to_be_defined_-_Eviden.

[84] DeChristopher LR. Perspective: the paradox in dietary advanced glycation end products research-the source of the serum and urinary advanced glycation end products is the intestines, not the food. Food Adv Nutr. 2017;8(5):679–83. 10.3945/an.117.016154. https://pubmed.ncbi.nlm.nih.gov/28916568/. PMID: 28916568; PMCID: PMC5593110. 28916568 10.3945/an.117.016154 PMC5593110

[85] van der Lugt T, Venema K, van Leeuwen S, Vrolijk MF, Opperhuizen A, Bast A. Gastrointestinal digestion of dietary advanced glycation endproducts using an in vitro model of the gastrointestinal tract (TIM-1). Food Funct. 2020;11(7):6297–307. 10.1039/d0fo00450b. https://pubmed.ncbi.nlm.nih.gov/32602872/. 32602872 10.1039/d0fo00450b

[86] Bains Y, Gugliucci A. Ilex paraguariensis and its main component chlorogenic acid inhibit fructose formation of advanced glycation endproducts with amino acids at conditions compatible with those in the digestive system. Fitoterapia. 2017;117:6–10. https://pubmed.ncbi.nlm.nih.gov/28012919/. 28012919 10.1016/j.fitote.2016.12.006

[87] Bains Y, Gugliucci A, Caccavello R. Advanced glycation endproducts form during ovalbumin digestion in the presence of fructose: inhibition by chlorogenic acid. Fitoterapia. 2017;120:1–5. https://pubmed.ncbi.nlm.nih.gov/28527897/. 28527897 10.1016/j.fitote.2017.05.003

[88] Martinez-Saez N, Fernandez-Gomez B, Cai W, Uribarri J, Dolores del Castillo M. In vitro formation of Maillard reaction products during simulated digestion of meal-resembling systems. In Food Research International. 2017. ISSN 0963–9969, 10.1016/j.foodres.2017.09.056. http://www.sciencedirect.com/science/article/pii/S0963996917306415. 10.1016/j.foodres.2017.09.056 30898355

[89] Schaefer EJ, Gleason JA, Dansinger ML. Dietary fructose and glucose differentially affect lipid and glucose homeostasis. J Nutr. 2009;139(6):1257S-1262S. 10.3945/jn.108.098186. Epub 2009 Apr 29. PMID: 19403705; PMCID: PMC2682989. Dietary fructose and glucose differentially affect lipid and glucose homeostasis - PubMed (nih.gov)). 19403705 10.3945/jn.108.098186 PMC2682989

[90] Nadkar MY, Jain VI. Serum uric acid in acute myocardial infarction. J Assoc Phys India. 2008;56:759–62. https://pubmed.ncbi.nlm.nih.gov/19263700/. 19263700

[91] Nakagawa T, Hu H, Zharikov S, Tuttle KR, Short RA, Glushakova O, Ouyang X, Feig DI, Block ER, Herrera-Acosta J, Patel JM, Johnson RJ. A causal role for uric acid in fructose-induced metabolic syndrome. Am J Physiol Renal Physiol. 2006;290(3):F625–31. 10.1152/ajprenal.00140.2005. Epub 2005 Oct 18. PMID: 16234313 A causal role for uric acid in fructose-induced metabolic syndrome - PubMed (nih.gov). 16234313 10.1152/ajprenal.00140.2005

[92] Theytaz F, de Giorgi S, Hodson L, Stefanoni N, Rey V, Schneiter P, Giusti V, Tappy L. Metabolic fate of fructose ingested with and without glucose in a mixed meal. Nutrients. 2014;6(7):2632–49. 10.3390/nu6072632. https://www.ncbi.nlm.nih.gov/pmc/articles/PMC4113761/. PMID: 25029210; PMCID: PMC4113761. 25029210 10.3390/nu6072632 PMC4113761

[93] The Nestle Company. Nestle raises the bar on ice cream with move to simpler ingredients. 2016. https://www.nestleusa.com/media/pressreleases/nestle-dreyers-ice-cream-simpler-ingredient-improvement.

[94] Chen Y, Zhu Y, Wu C, Lu A, Deng M, Yu H, Huang C, Wang W, Li C, Zhu Q, Wang L. Gut dysbiosis contributes to high fructose-induced salt-sensitive hypertension in Sprague-Dawley rats. Nutrition. 2020;75:110766. 10.1016/j.nut.2020.110766. https://pubmed.ncbi.nlm.nih.gov/32305658/. Epub 2020 Feb 14. Erratum in: Nutrition. 2020 May 28;:110845. PMID: 32305658. 32305658 10.1016/j.nut.2020.110766

[95] Yan Q, Gu Y, Li X, Yang W, Jia L, Chen C, Han X, Huang Y, Zhao L, Li P, Fang Z, Zhou J, Guan X, Ding Y, Wang S, Khan M, Xin Y, Li S, Ma Y. Alterations of the gut microbiome in hypertension. Front Cell Infect Microbiol. 2017;7:381. 10.3389/fcimb.2017.00381. https://pubmed.ncbi.nlm.nih.gov/28884091/. PMID: 28884091; PMCID: PMC5573791. 28884091 10.3389/fcimb.2017.00381 PMC5573791

[96] Packard CJ, Boren J, Taskinen MR. Causes and consequences of hypertriglyceridemia. Front Endocrinol (Lausanne). 2020;14(11):252. 10.3389/fendo.2020.00252. https://pubmed.ncbi.nlm.nih.gov/32477261/. PMID: 32477261; PMCID: PMC7239992. 10.3389/fendo.2020.00252 PMC7239992 32477261

[97] Yoo JY, Sniffen S, McGill Percy KC, Pallaval VB, Chidipi B. Gut dysbiosis and immune system in Atherosclerotic Cardiovascular Disease (ACVD). Microorganisms. 2022;10(1):108. 10.3390/microorganisms10010108. https://www.ncbi.nlm.nih.gov/pmc/articles/PMC8780459/. PMID: 35056557; PMCID: PMC8780459. 35056557 10.3390/microorganisms10010108 PMC8780459

[98] Li M, Gong W, Wang S, Li Z. Relationship between high fructose corn syrup sweetened drinks, diet soft drinks, and serum sodium: NHANES 2003–2006. Nutr J. 2022;21(1):76. 10.1186/s12937-022-00832-7. https://pubmed.ncbi.nlm.nih.gov/36581871/. PMID: 36581871; PMCID: PMC9798711. 36581871 10.1186/s12937-022-00832-7 PMC9798711

[99] Jensen ET, Bertoni AG, Crago OL, Hoffman KL, Wood AC, Arzumanyan Z, Lam LK, Roll K, Sandow K, Wu M, Rich SS, Rotter JI, Chen YI, Petrosino JF, Goodarzi MO. Rationale, design and baseline characteristics of the Microbiome and Insulin Longitudinal Evaluation Study (MILES). Diabetes Obes Metab. 2020;22(11):1976–84. 10.1111/dom.14145. https://pubmed.ncbi.nlm.nih.gov/32687239/. Epub 2020 Aug 20. PMID: 32687239; PMCID: PMC8444996. 32687239 10.1111/dom.14145 PMC8444996

[100] Aslamy A, Wood AC, Jensen ET, Bertoni AG, Sheridan PA, Wong KE, Ramesh G, Rotter JI, Chen YI, Goodarzi MO. Increased plasma branched short-chain fatty acids and improved glucose homeostasis: the Microbiome and Insulin Longitudinal Evaluation Study (MILES). Diabetes. 2024;73(3):385–90. 10.2337/db23-0401. https://pubmed.ncbi.nlm.nih.gov/37992186/. PMID: 37992186; PMCID: PMC10882143. 37992186 10.2337/db23-0401 PMC10882143

[101] Tian M, Li R, Shan Z, Wang DW, Jiang J, Cui G. Comparison of Apolipoprotein B/A1 ratio, Framingham risk score and TC/HDL-c for predicting clinical outcomes in patients undergoing percutaneous coronary intervention. Lipids Health Dis. 2019;18(1):202. 10.1186/s12944-019-1144-y5. https://pubmed.ncbi.nlm.nih.gov/31744496/. PMID: 31744496; PMCID: PMC6864950. 31744496 10.1186/s12944-019-1144-y5 PMC6864950

[102] Lau K, Srivatsav V, Rizwan A, Nashed A, Liu R, Shen R, Akhtar M. Bridging the gap between gut microbial dysbiosis and cardiovascular diseases. Nutrients. 2017;9(8):859. 10.3390/nu9080859. https://www.ncbi.nlm.nih.gov/pmc/articles/PMC5579652/. PMID: 28796176; PMCID: PMC5579652. 28796176 10.3390/nu9080859 PMC5579652

[103] Chen PB, Black AS, Sobel AL, et al. Directed remodeling of the mouse gut microbiome inhibits the development of atherosclerosis [published online ahead of print, 2020 Jun 15]. Nat Biotechnol. 2020. 10.1038/s41587-020-0549-5. https://pubmed.ncbi.nlm.nih.gov/32541956/. 10.1038/s41587-020-0549-5 PMC7641989 32541956

[104] Zhang Q, Ames JM, Smith RD, Baynes JW, Metz TO. A perspective on the Maillard reaction and the analysis of protein glycation by mass spectrometry: probing the pathogenesis of chronic disease. J Proteome Res. 2009;8(2):754–69. 10.1021/pr800858h. https://pubs.acs.org/doi/10.1021/pr800858h. 19093874 10.1021/pr800858h PMC2642649

[105] Liu Z, Wang Y, Zhu H, Qiu C, Guan G, Wang J, Guo Y. Matrine blocks AGEs- induced HCSMCs phenotypic conversion via suppressing Dll4-Notch pathway. Eur J Pharmacol. 2018;835:126–31. 10.1016/j.ejphar.2018.07.051. https://pubmed.ncbi.nlm.nih.gov/30063915/. Epub 2018 Jul 29. PMID: 30063915. 30063915 10.1016/j.ejphar.2018.07.051

[106] Chen Y, Guo TL. Dietary advanced glycation end-products elicit toxicological effects by disrupting gut microbiome and immune homeostasis. J Immunotoxicol. 2021;18(1):93–104. 10.1080/1547691X.2021.1959677. https://pubmed.ncbi.nlm.nih.gov/34436982/. PMID: 34436982. 34436982 10.1080/1547691X.2021.1959677 PMC9885815

[107] Cavero-Redondo I, Soriano-Cano A, Álvarez-Bueno C, Cunha PG, Martínez-Hortelano JA, Garrido-Miguel M, Berlanga-Macías C, Martínez-Vizcaíno V. Skin autofluorescence-indicated advanced glycation end products as predictors of cardiovascular and all-cause mortality in high-risk subjects: a systematic review and meta-analysis. J Am Heart Assoc. 2018;7(18):e009833. 10.1161/JAHA.118.009833. https://pubmed.ncbi.nlm.nih.gov/30371199/. PMID: 30371199; PMCID: PMC6222966. 30371199 10.1161/JAHA.118.009833 PMC6222966

[108] Chiang KH, Chen JW, Huang SS, Leu HB, Lin SJ, Huang PH. The ratio of AGE to sRAGE independently associated with albuminuria in hypertensive patients. BMC Endocr Disord. 2018;18(1):84. 10.1186/s12902-018-0306-7. https://pubmed.ncbi.nlm.nih.gov/30424768/. PMID: 30424768; PMCID: PMC6234555. 30424768 10.1186/s12902-018-0306-7 PMC6234555

[109] Keller A, O’Reilly EJ, Malik V, Buring JE, Andersen I, Steffen L, Robien K, Männistö S, Rimm EB, Willett W, Heitmann BL. Substitution of sugar-sweetened beverages for other beverages and the risk of developing coronary heart disease: results from the Harvard Pooling Project of Diet and Coronary Disease. Prev Med. 2020;131:105970. 10.1016/j.ypmed.2019.105970. https://pubmed.ncbi.nlm.nih.gov/31883872/. Epub 2019 Dec 26. PMID: 31883872. 31883872 10.1016/j.ypmed.2019.105970

[110] Eshak ES, Iso H, Kokubo Y, Saito I, Yamagishi K, Inoue M, Tsugane S. Soft drink intake in relation to incident ischemic heart disease, stroke, and stroke subtypes in Japanese men and women: the Japan Public Health Centre-based study cohort I. Am J Clin Nutr. 2012;96(6):1390–7. 10.3945/ajcn.112.037903. https://pubmed.ncbi.nlm.nih.gov/23076619/. Epub 2012 Oct 17. PubMed PMID: 23076619. 23076619 10.3945/ajcn.112.037903

[111] Yang Q, Zhang Z, Gregg EW, Flanders WD, Merritt R, Hu FB. Added sugar intake and cardiovascular diseases mortality among US adults. JAMA Intern Med. 2014;174(4):516–24. 10.1001/jamainternmed.2013.13563. https://pubmed.ncbi.nlm.nih.gov/24493081/. PubMed PMID: 24493081. 24493081 10.1001/jamainternmed.2013.13563 PMC10910551

[112] Haley S. Sugars and Sweeteners Outlook. United States Department of Agriculture. SSS-M-270. 2011. p. 6–9. https://www.ers.usda.gov/webdocs/outlooks/39259/19597_sssm270_1_.pdf?v=7981.6. Accessed 6 Mar 2021.

[113] Haley S. Sugars and Sweeteners Outlook. United States Department of Agriculture. SSM-M-286. 2012. p. 16–19. https://www.ers.usda.gov/webdocs/outlooks/39309/28794_sssm286.pdf?v=8469.7. Accessed 6 Mar 2021.

[114] Strom S. U.S. Cuts Estimate of Sugar Intake. The New York Times. 2012. http://www.nytimes.com/2012/10/27/business/us-cuts-estimate-of-sugar-intake-of-typical-american.html. Accessed 19 May 2024.

[115] Heart Failure Before Age 50 More Common in Black People. National Institutes of Health. 2009. https://www.nih.gov/news-events/nih-research-matters/heart-failure-age-50-more-common-black-people.

[116] Daniels LA. Coke, Pepsi to Use More Corn Syrup. Section D. Page 6. Now in the New York Times Archives. 1984. https://www.nytimes.com/1984/11/07/business/coke-pepsi-to-use-more-corn-syrup.html.

[117] Bray GA. Energy and fructose from beverages sweetened with sugar or high-fructose corn syrup pose a health risk for some people. Adv Nutr. 2013;4(2):220–5. 10.3945/an.112.002816. https://pubmed.ncbi.nlm.nih.gov/23493538/. Review. PubMed PMID: 23493538; PubMed Central PMCID: PMC3649102. 23493538 10.3945/an.112.002816 PMC3649102

[118] Bray GA, Nielsen SJ, Popkin BM. Consumption of high-fructose corn syrup in beverages may play a role in the epidemic of obesity. Am J Clin Nutr. 2004;79(4):537–43. https://pubmed.ncbi.nlm.nih.gov/15051594/. Review. Erratum in: Am J Clin Nutr. 2004 Oct;80(4):1090. PubMed PMID: 15051594. 15051594 10.1093/ajcn/79.4.537

[119] Blackwell. Canadian researchers have received hundreds of thousands from soft-drink makers and the sugar industry. National Post. 2015. https://nationalpost.com/health/canadian-researchers-have-received-hundreds-of-thousands-from-soft-drink-makers-and-the-sugar-industry.

[120] Kearns CE, Schmidt LA, Glantz SA. Sugar industry and coronary heart disease research. An internal analysis of internal industry documents. JAMA Intern Med. 2016. 10.1001/jamainternmed.2016.5394. https://www.ncbi.nlm.nih.gov/pubmed/27617709. 10.1001/jamainternmed.2016.5394 PMC5099084 27617709

[121] Fox A. Complaint for damages and injunctive relief for false advertising in the violation of (1) the Lantham Act (15 U.S.C. & 1125(a)), and (2) California’s Unfair Competition Law (Cal. Bus. & Prof. Code & 17200, Et. seq. Filed 4/22/2011. https://www.scribd.com/document/288646633/Western-Sugar-Cooperative-et-al-v-Archer-Daniels-Midland-Company-et-al-Complaint.

[122] Fox A. Second amended complaint for damages and injunctive relief for false advertising in violation of the Lantham Act. Filed 11/18/2011. https://www.scribd.com/document/94868291/2d-Am-Complaint-Western-Sugar-Coop-v-Archer-Daniels-Midland.

[123] White JS, Hobbs LJ, Fernandez S. Fructose content and composition of commercial HFCS-sweetened carbonated beverages. Int J Obes (Lond). 2015;39(1):176–82. 10.1038/ijo.2014.73. https://www.nature.com/articles/ijo201473. Epub 2014 May 6. Review. PubMed PMID: 24798032; PubMed Central PMCID: PMC4285619. 24798032 10.1038/ijo.2014.73 PMC4285619

[124] Tuck CJ, Ross LA, Gibson PR, Barrett JS, Muir JG. Adding glucose to food and solutions to enhance fructose absorption is not effective in preventing fructose-induced functional gastrointestinal symptoms: randomised controlled trials in patients with fructose malabsorption. J Hum Nutr Diet. 2017;30(1):73–82. 10.1111/jhn.12409. https://pubmed.ncbi.nlm.nih.gov/27600184/. 27600184 10.1111/jhn.12409

[125] Hill JO, Sidney S, Lewis CE, Tolan K, Scherzinger AL, Stamm ER. Racial differences in amounts of visceral adipose tissue in young adults: the CARDIA (Coronary Artery Risk Development in Young Adults) study. Am J Clin Nutr. 1999;69(3):381–7. 10.1093/ajcn/69.3.381. https://pubmed.ncbi.nlm.nih.gov/10075320/. PMID: 10075320. 10075320 10.1093/ajcn/69.3.381

